# Identification and characterization of nuclear genes involved in photosynthesis in *Populus*

**DOI:** 10.1186/1471-2229-14-81

**Published:** 2014-03-27

**Authors:** Bowen Wang, Qingzhang Du, Xiaohui Yang, Deqiang Zhang

**Affiliations:** 1 National Engineering Laboratory for Tree Breeding, College of Biological Sciences and Technology, Beijing Forestry University, Beijing 100083, P. R. China; 2 Key Laboratory of Genetics and Breeding in Forest Trees and Ornamental Plants, Ministry of Education, College of Biological Sciences and Technology, Beijing Forestry University, Beijing 100083, P. R. China

## Abstract

**Background:**

The gap between the real and potential photosynthetic rate under field conditions suggests that photosynthesis could potentially be improved. Nuclear genes provide possible targets for improving photosynthetic efficiency. Hence, genome-wide identification and characterization of the nuclear genes affecting photosynthetic traits in woody plants would provide key insights on genetic regulation of photosynthesis and identify candidate processes for improvement of photosynthesis.

**Results:**

Using microarray and bulked segregant analysis strategies, we identified differentially expressed nuclear genes for photosynthesis traits in a segregating population of poplar. We identified 515 differentially expressed genes in this population (*FC* ≥ 2 or *FC ≤* 0.5, *P* < 0.05), 163 up-regulated and 352 down-regulated. Real-time PCR expression analysis confirmed the microarray data. Singular Enrichment Analysis identified 48 significantly enriched GO terms for molecular functions (28), biological processes (18) and cell components (2). Furthermore, we selected six candidate genes for functional examination by a single-marker association approach, which demonstrated that 20 SNPs in five candidate genes significantly associated with photosynthetic traits, and the phenotypic variance explained by each SNP ranged from 2.3% to 12.6%. This revealed that regulation of photosynthesis by the nuclear genome mainly involves transport, metabolism and response to stimulus functions.

**Conclusions:**

This study provides new genome-scale strategies for the discovery of potential candidate genes affecting photosynthesis in *Populus*, and for identification of the functions of genes involved in regulation of photosynthesis. This work also suggests that improving photosynthetic efficiency under field conditions will require the consideration of multiple factors, such as stress responses.

## Background

Addressing global concerns regarding crop yield and fossil fuel energy will require novel strategies. Photosynthesis harnesses solar energy, produces carbohydrates and biomaterials and sustains the carbon/oxygen cycle; supercharging photosynthesis may help to address these concerns and meet increasing demands for food, fuel, and biomaterials [1,2]. Improving photosynthesis in annual, herbaceous crops would provide substantial benefits, as more than 90% of crop biomass derives from photosynthetic products [3]. The woody tissues of trees make up roughly 20% of the total terrestrial carbon storage [4,5], and also derive from photosynthetic products. Therefore, improving photosynthesis in perennial, woody crops would improve carbon fixation, biomaterials production, and biofuels efficiency.

Despite its importance, photosynthesis has inefficiencies: plants typically convert only 2% to 4% of the available solar energy into plant growth [6]. The gap between the real and potential photosynthetic rate under field conditions suggests that photosynthesis could be improved to increase crop yields and improve the economics of biofuel production. However, the complex process of photosynthesis includes many potential target reactions, such as light harvesting, carbon fixation, photophosphorylation and electron transport [7]. Potential ways to improve photosynthesis include directly improving the mechanism of photosynthesis itself [7], or exploring and analyzing the complexity of photosynthetic regulation in the field to improve photosynthesis from a whole-plant perspective. Studies exploring improvements to the mechanism of photosynthesis have been widely reported [2,7]. Current proposals to improve photosynthesis mainly include improving the performance of Rubisco [1], decreasing photorespiration [8] and adding new biosynthetic pathways to increase the flow of carbon into useful products [9]. However, efforts to improve chloroplast-related processes may be constrained by known and unknown factors. For example, increasing photosynthetic rates by engineering photosynthetic pathways may overburden the capacity of the transport systems inside and outside of the chloroplast [10]. Exploring and analyzing the complexity of photosynthetic regulation in field conditions may identify these constraints and allow researchers to bypass potential difficulties.

The role of the nucleus should command particular attention among the mechanisms regulating photosynthesis. The chloroplast, as the site of photosynthesis, requires adaptive changes in the expression levels of nuclear genes to respond to endogenous and environmental stimuli; a complex signaling network regulates these changes in gene expression [11-13]. Chloroplast and chloroplast-related processes have been studied in detail [14]. The higher plant nuclear genome encodes about 2,090 predicted chloroplast proteins (http://rarge.psc.riken.jp/chloroplast/), and chloroplast metabolism is tightly integrated with the rest of the cell (http://www.grenoble.prabi.fr/at_chloro/) [15,16]. Hence, the nucleus plays a crucial role in maintaining normal function of the chloroplast [17,18].

Nuclear genes provide potential targets for improving photosynthetic efficiency. For example, Rubisco, the enzyme that catalyzes the reaction of CO_2_ into ribulose 1,5-bisphosphate (RuBP), is a prime target for genetic engineering to improve photosynthetic efficiency [19], but the locations of *rbc*L in the chloroplast genome and *Rbc*S in the nuclear genome have hindered this approach [20]. Various other biological processes regulated by the nucleus [21] also may be useful targets for improving photosynthesis, such as plant morphology [22], photoprotection [23], and the regulation of photosynthesis by the sophisticated balance of energy [24] and redox [25]. However, the nuclear genes involved in the regulation of photosynthesis remain poorly understood, especially in trees, despite the importance of nuclear genes in all photosynthesis-related processes. Trees are extremely long-lived and generate woody biomass derived from plant cell wall lignocellulose. So, in trees, the mechanisms of nuclear regulation of photosynthesis may be even more complicated than in herbaceous annuals.

Studies of the regulation of photosynthesis have mainly focused on the influence of environmental factors or the interaction with other biological processes. Most stresses, including biotic [26], chilling [27], drought [28] and salt [29] stress, repress photosynthesis. Ecosystem respiration is tightly coupled with canopy photosynthesis [30]. Also, transport mechanisms and efficiency influence photosynthetic productivity by relieving product inhibition and contribute to plant vigor by controlling source/sink relationships and biomass partitioning [31]. However, little is known about the role of nuclear genes in photosynthesis. *Populus*, as a model for long-lived woody perennials [32], can be used to study the molecular basis of regulation of photosynthesis at the genome scale in woody plants. Based on the high-quality, annotated genome sequence of *Populus trichocarpa*[33], emerging research has investigated biological functions in woody plants including wood formation, seasonality, flowering control, dormancy release, senescence, biotic interactions and abiotic stress responses [34].

In the present study, we used a segregating population of poplars with different net photosynthetic rates (*Pn*), to identify and characterize nuclear genes involved in photosynthesis in trees, by combining microarray techniques and bulked segregant analysis (BSA). To our knowledge, this is the first systematic, genome-wide investigation of differentially expressed genes in pools of progeny with different photosynthetic rates in *Populus*. We identified 515 differentially expressed genes in this population according to the fold change (FC) of gene expression (*FC* ≥ 2 or *FC ≤* 0.5, *P* < 0.05). Also, we outlined a putative gene network in the *Populus* nuclear genome for regulation of photosynthesis. Furthermore, the differentially expressed genes identified here may be suitable targets for biotechnological manipulation to improve photosynthesis.

## Results

### Phenotypic variation and correlation

For this study, we used a linkage mapping population of 1200 F_1_ progeny, from the cross of two elite poplar parents, the female hybrid clone “YX01” (*P. alba* × *P. glandulossa*) and the male clone “LM50” (*P. tomentosa*). We first characterized the variation in photosynthetic characteristics, including *Pn*, stomatal conductance (*Cond*), intercellular CO_2_ concentration (*Ci*) and transpiration rate (*Trmmol*), in this population. The photosynthetic characteristics *Pn*, *Cond*, *Ci*, and *Trmmol* of the 1200 F_1_ individuals had a continuous distribution indicating that all measured traits were quantitatively inherited and each trait showed significant differences in the population (Additional file 1: Figure S1). Means of the four photosynthetic characteristics were: 13.360 ± 0.116 (mean ± SE) μmol · m^−2^ · s^−1^ (*Pn*), 0.225 ± 0.003 mol · m^−2^ · s^−1^ (*Cond*), 243.203 ± 2.026 μmol · mol^−1^(*Ci*), and 4.571 ± 0.047 g · m^−2^ · h^−1^ (*Trmmol*) and their coefficients of variation (CV) ranged from 27.755% to 49.333%. The CV of *Pn* and *Ci* were similar, at 28.915% and 27.755%, respectively (Additional file 2: Table S1). We also measured three growth traits and four wood property traits. CVs of growth traits including tree height (*H*), diameter at chest height (*D*) and stem volume (*V*) were 25.403%, 29.464% and 78.630% respectively. For *D*, we define “chest height” as 1.3 meters above the ground. Wood traits showed lower CVs; for example, the CV of holocellulose content was the smallest, at just 1.941%. Descriptive statistics of the trait distributions are presented in Additional file 2: Table S1. The broad sense heritability (h^2^) was estimated for each trait (Additional file 3: Table S2) ranged from 65.76% to 99.97%. *Pn* had the highest h^2^ value, followed by *Cond*, *V*, *D*, *Trmmol*, *H*, *Ci*, Lignin content, microfiber angle and α-cellulose content. The h^2^ of holocellulose content was lowest. Higher h^2^ values were observed in photosynthetic characteristics and growth traits (*D, H, V*).

The phenotypic and genetic correlations between photosynthesis and growth and wood quality traits measured were summarized in Table 1. The genetic correlations were in general stronger than the respective phenotypic correlations. As expected, the growth traits displayed significant positive phenotypic and genetic correlations with photosynthetic traits in the population (Table 1). For example, *Pn* was significantly positively correlated with all growth traits (*P* < 0.01) including *H, D* and *V.* Genetic correlations ranged from 0.312 to 0.332, suggesting that photosynthesis may have a direct correlation with plant growth. However, we did not observe a significant genetic correlation between the photosynthetic traits (*Pn*, *Cond*, *Ci* and *Trmmol*) and three wood chemical traits (lignin content, holocellulose content and α-cellulose content). This suggested that photosynthesis may only indirectly influence wood chemical properties, and indicates that these biological pathways are very complex. We observed significant correlations between wood traits (*P* < 0.01); for example, lignin content was negatively correlated with holocellulose content (*P* < 0.05) and α-cellulose content (*P* < 0.05). Conversely, lignin content was positively correlated with microfiber angle (*P* < 0.01) (Table 1).

**Table 1:** Phenotypic (above the diagonal) and genetic (below the diagonal) correlations for photosynthetic characteristics and wood traits in the linkage mapping population (n = 300)

	***Pn***	***Cond***	***Ci***	***Trm***	***D***	***H***	***V***	***MFA***	***Hol***	***α-cel***	***Lig***
*Pn*	1	0.624**	0.066**	0.374**	0.330**	0.322**	0.310**	−0.109**	−0.011	0.007	−0.086**
*Cond*	0.626**	1	0.729**	0.732**	0.101**	0.075*	0.058	−0.042	0.016	−0.035	−0.082*
*Ci*	0.067	0.738**	1	0.756**	−0.079*	−0.098**	−0.107**	0.041	0.047	−0.032	−0.060
*Trm*	0.376**	0.739**	0.770**	1	0.128**	0.114**	0.089**	0.044	0.038	0.027	0.007
*D*	0.332**	0.101	−0.080	0.130*	1	0.722**	0.938**	−0.035	0.026	0.147**	−0.066*
*H*	0.325**	0.075	−0.099	0.118*	0.732**	1	0.772**	−0.064	0.061	0.155**	−0.025
*V*	0.312**	0.036	−0.108	0.091	0.940**	0.776**	1	−0.040	0.033	0.144**	−0.058
*MFA*	−0.114*	−0.043	0.047	0.050	−0.037	−0.070	−0.043	1	−0.096**	−0.180**	0.151**
*Hol*	−0.023	0.035	0.084	0.074	0.050	0.130	0.064	−0.137*	1	0.200**	−0.080*
*α-cel*	0.008	−0.038	−0.030	0.027	0.155**	0.164**	0.153**	−0.209**	0.455**	1	−0.367*
*Lig*	−0.088	−0.084	−0.063	0.006	−0.068	−0.027	−0.060	0.160**	−0.164*	−0.398**	1

**P ≤ 0.01; *P ≤ 0.05. *P*-value shows the credibility of correlation.

*Pn*, photosynthetic rate; *Cond*, conductance to H_2_O; *Ci*, intercellular CO_2_ concentration; *Trm*, transpiration rate; *MFA*, microfiber angle; *H*, tree height; *D*, diameter at chest height; *V*, stem volume; *Hol*, holocellulose; *α-cel*, α-cellulose; *Lig*, lignin content.

### Identification of differentially expressed genes

To find nuclear genes involved in photosynthesis, we compared the gene expression profiles of pools constructed by bulked segregant analysis (BSA) [35]. Specifically, we identified plants with very high and very low photosynthetic rate (*Pn*). Plants with high or low *Pn* were pooled and compared. Microarray-based comparison of the FC of gene expression between the pools with high and low photosynthetic efficiency showed that only 0.84% of all probes (515 out of 61,313) showed a significant difference (*FC* ≥ 2 or *FC ≤* 0.5, *P* < 0.05). Of these, 162 probes were up-regulated (see Additional file 4) and 353 were down-regulated (see Additional file 5) in plants with high *Pn*, compared to the plants with low *Pn*. The fact that most of the significantly differentially expressed genes were repressed in plants with high *Pn* (68.54%) may be explained because plants under field conditions rarely realize their maximum photosynthetic potential [7]. Of the differentially expressed genes, 74 were annotated as having no known function (unknown proteins, hypothetical proteins, etc.) and of these, 30 (40.54%) were up-regulated (see Additional file 4), suggesting that they were bona fide candidates for novel, up-regulated photosynthesis genes.

### Verification of the differentially expressed genes identified by microarray

**Figure 1:**
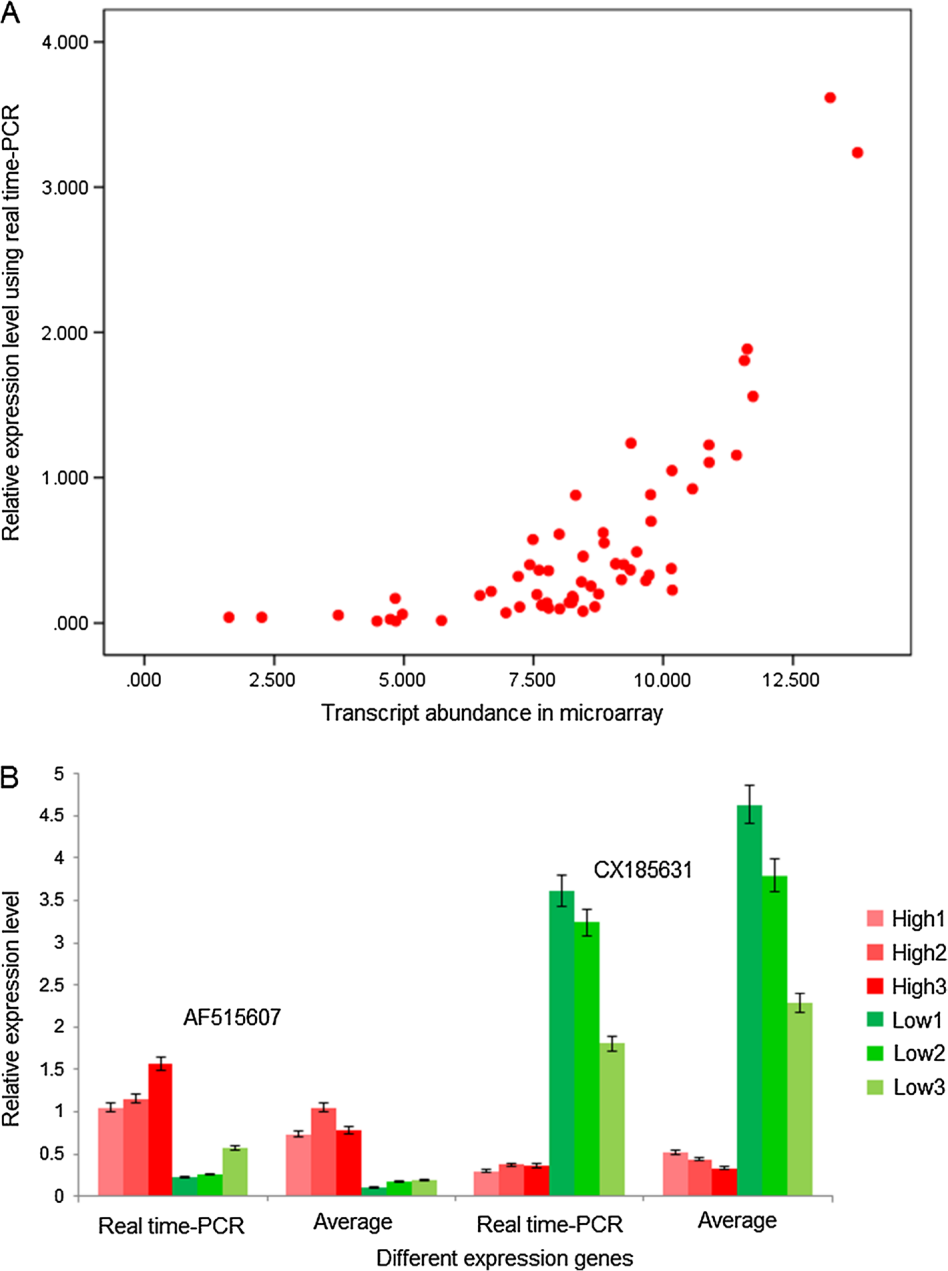
**Validation of selected candidate genes using real time-PCR.** We selected ten genes for validation by real time-PCR: CF936190, AF515607, CK089075, CV273041 and CV260219A are the up-regulated genes, and AJ780277, CX183751, CX185631, CV260015 and DN487027 are the down-regulated genes in high photosynthetic rate gene pools. **(A)** The scatter plot show the relationship between transcript abundance of candidate genes in microarray and the relative expression level using real time-PCR, for which the template was the mixed cDNA of BSA (bulked segregant analysis) pools. **(B)** The relative expression level using real time-PCR, for which the template was the mixed cDNA of BSA pools and the templates were from the individuals used for constructing BSA pools. Error bars represent the standard deviation. Real time-PCR expression analysis with ANOVA indicated significant differences between the groups with high and low photosynthetic rate (Additional file 7: Table S4), generally consistent with the expression patterns identified by the microarray. The trends are consistent between Real time-PCR expression measured in the gene pool and relative individuals (constructing these gene pools) in general (r = 0.939).

We confirmed the transcriptional regulation measured by microarray in a biologically independent experiment using real time-PCR (RT-PCR) with gene-specific primers (Additional file 6: Table S3). We chose ten differentially expressed genes, including five up- and five down-regulated genes, in plants with high *Pn*, compared to plants with low *Pn*, to assess the accuracy of the microarray data. We selected the set of genes to have functions covering all putative biological progresses affecting photosynthesis, including: hormone signaling, carbohydrate metabolism, response to stress, response to redox, transcript regulation and transport (Additional file 6: Table S3). RT-PCR expression analysis and *t*-test analysis (Additional file 7: Table S4) indicated significant differences between the groups with high and low photosynthetic rate, consistent with the microarray data (Figure 1A), and a Pearson’s product–moment correlation test showed a positive correlation (r = 0.722) between the microarray and RT-PCR data. In addition, the expression pattern in high and low *Pn* pools was highly consistent for individuals within each pool (Pearson’s product–moment correlation, r = 0.939) (Figure 1B, Additional file 7: Table S4). The results indicated that the microarray experiments in this study are sufficiently reliable for identification of nuclear genes involved in photosynthesis in *Populus*.

### GO analysis and co-expression patterns

**Figure 2:**
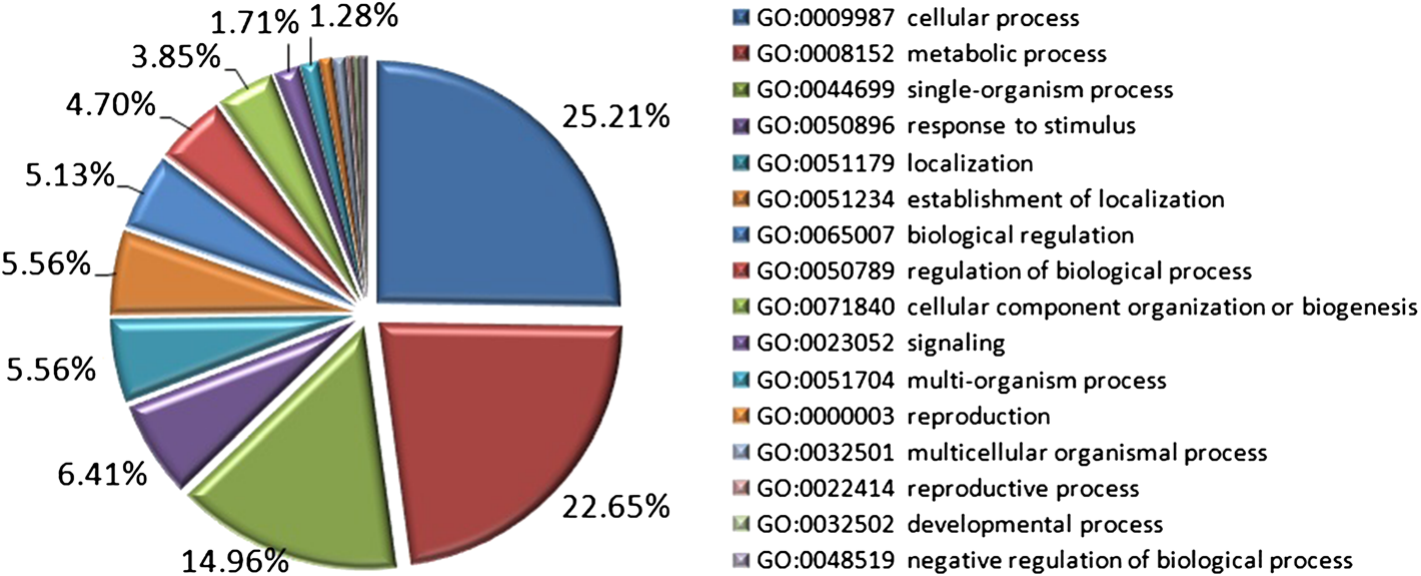
**Functional classification of GO terms of 515 differentially expressed genes.** In biological process, CateGOrizer grouped GO terms into 16 categories. The data for GO categories that represented less than 1% of the differentially-expressed genes are not shown. The most prevalent GO slim categories are cellular process (25.21%), metabolic process (22.65%) and single-organism process (14.96%), suggesting a high degree of basic metabolic activity in the regulation of nuclear genes in photosynthesis.

To functionally characterize the differentially expressed genes, we used CateGOrizer (http://www.animalgenome.org/tools/catego/) to analyze the GO terms for all the identified genes. For biological process (GO:0008150), the most prevalent GO slim categories were cellular process (25.21%), metabolic process (22.65%) and single-organism process (14.96%), suggesting a high degree of basic metabolic activity in the regulation of nuclear genes in photosynthesis (Figure 2). To identify significantly enriched GO terms, we used Singular Enrichment Analysis (SEA) on the differentially expressed genes. We identified 48 significantly enriched GO terms in: molecular functions (28), biological processes (18) and cell components (2) (Table 2). Eighteen of the enriched terms specify various metabolic or catabolic processes, for categories based on biological process (Table 2). The terms mostly relate to energy metabolism and biomass formation (e.g., aminoglycan, carbohydrate, polysaccharide, cell wall macromolecule). This suggested that metabolism plays an important role in regulation of nuclear genes in photosynthesis. For molecular functions, we found enrichment of terms involved in transcription, such as transcription factor activity (GO:0003700, *P* = 0.0019) and sequence-specific DNA binding (GO:0043565, *P* = 0.00015). Also, SEA showed a significant difference between up- and down-regulated terms (Additional file 8: Table S5). GO terms of the up-regulated genes were enriched for cell components including cell wall (GO: 0005618, *P* = 2.90E-06) and external encapsulating structure (GO: 0030312, *P* = 6.00E-07). By contrast, GO terms of the down-regulated genes were enriched in molecular functions. This analysis revealed that the nuclear genome participated in photosynthesis via different processes.

**Table 2:** Gene ontology enrichment analysis of the differentially expressed genes

**Category**	**GO term**	**Description**	**Number in input list**	**Number in BG/Ref**	**p-value**	**FDR**
Biological process	GO:0006030	chitin metabolic process	5	23	2.00E-06	0.00018
	GO:0006032	chitin catabolic process	5	23	2.00E-06	0.00018
	GO:0008152	metabolic process	143	9587	1.10E-06	0.00018
	GO:0006026	aminoglycan catabolic process	5	23	2.00E-06	0.00018
	GO:0006022	aminoglycan metabolic process	5	24	2.60E-06	0.00018
	GO:0005975	carbohydrate metabolic process	23	867	1.40E-05	0.00081
	GO:0005976	polysaccharide metabolic process	9	156	1.90E-05	0.00095
	GO:0044036	cell wall macromolecule metabolic process	6	62	2.60E-05	0.001
	GO:0016998	cell wall macromolecule catabolic process	6	61	2.30E-05	0.001
	GO:0071554	cell wall organization or biogenesis	9	171	3.80E-05	0.0014
	GO:0000272	polysaccharide catabolic process	5	42	4.40E-05	0.0014
	GO:0044238	primary metabolic process	106	7479	9.10E-05	0.0027
	GO:0044092	negative regulation of molecular function	5	71	0.00054	0.014
	GO:0043086	negative regulation of catalytic activity	5	71	0.00054	0.014
	GO:0043170	macromolecule metabolic process	83	5955	0.0006	0.014
	GO:0016052	carbohydrate catabolic process	6	117	0.00084	0.019
	GO:0055114	oxidation reduction	18	857	0.0015	0.032
	GO:0009056	catabolic process	10	359	0.0023	0.045
Molecular	GO:0004553	hydrolase activity, hydrolyzing O-glycosyl compounds	20	477	5.10E-08	1.00E-05
Function	GO:0004857	enzyme inhibitor activity	13	188	3.60E-08	1.00E-05
	GO:0016798	hydrolase activity, acting on glycosyl bonds	20	505	1.30E-07	1.70E-05
	GO:0003824	catalytic activity	143	9307	2.70E-07	2.70E-05
	GO:0016787	hydrolase activity	56	2834	6.90E-07	5.40E-05
	GO:0004568	chitinase activity	5	23	2.00E-06	0.00011
	GO:0008061	chitin binding	5	24	2.60E-06	0.00011
	GO:0001871	pattern binding	5	24	2.60E-06	0.00011
	GO:0030247	polysaccharide binding	5	24	2.60E-06	0.00011
	GO:0030234	enzyme regulator activity	13	325	1.60E-05	0.00063
	GO:0030246	carbohydrate binding	12	312	4.90E-05	0.0017
	GO:0016491	oxidoreductase activity	36	1867	7.50E-05	0.0025
	GO:0043565	sequence-specific DNA binding	16	578	0.00015	0.0046
	GO:0030599	pectinesterase activity	7	137	0.00033	0.0091
	GO:0004866	endopeptidase inhibitor activity	5	67	0.00042	0.01
	GO:0030414	peptidase inhibitor activity	5	67	0.00042	0.01
	GO:0004713	protein tyrosine kinase activity	27	1395	0.00048	0.011
	GO:0005506	iron ion binding	15	584	0.00052	0.011
	GO:0004091	carboxylesterase activity	8	204	0.00073	0.015
	GO:0042802	identical protein binding	5	79	0.00088	0.017
	GO:0008236	serine-type peptidase activity	8	214	0.001	0.018
	GO:0017171	serine hydrolase activity	8	214	0.001	0.018
	GO:0020037	heme binding	13	504	0.0011	0.018
	GO:0046906	tetrapyrrole binding	13	504	0.0011	0.018
	GO:0043169	cation binding	39	2449	0.0015	0.021
	GO:0046872	metal ion binding	39	2445	0.0014	0.021
	GO:0043167	ion binding	39	2449	0.0015	0.021
	GO:0003700	transcription factor activity	17	805	0.0019	0.027
Cell component	GO:0030312	external encapsulating structure	13	166	8.20E-09	4.10E-07
	GO:0005618	cell wall	9	136	6.20E-06	0.00015

*FDR* false discovery rate.

**Figure 3:**
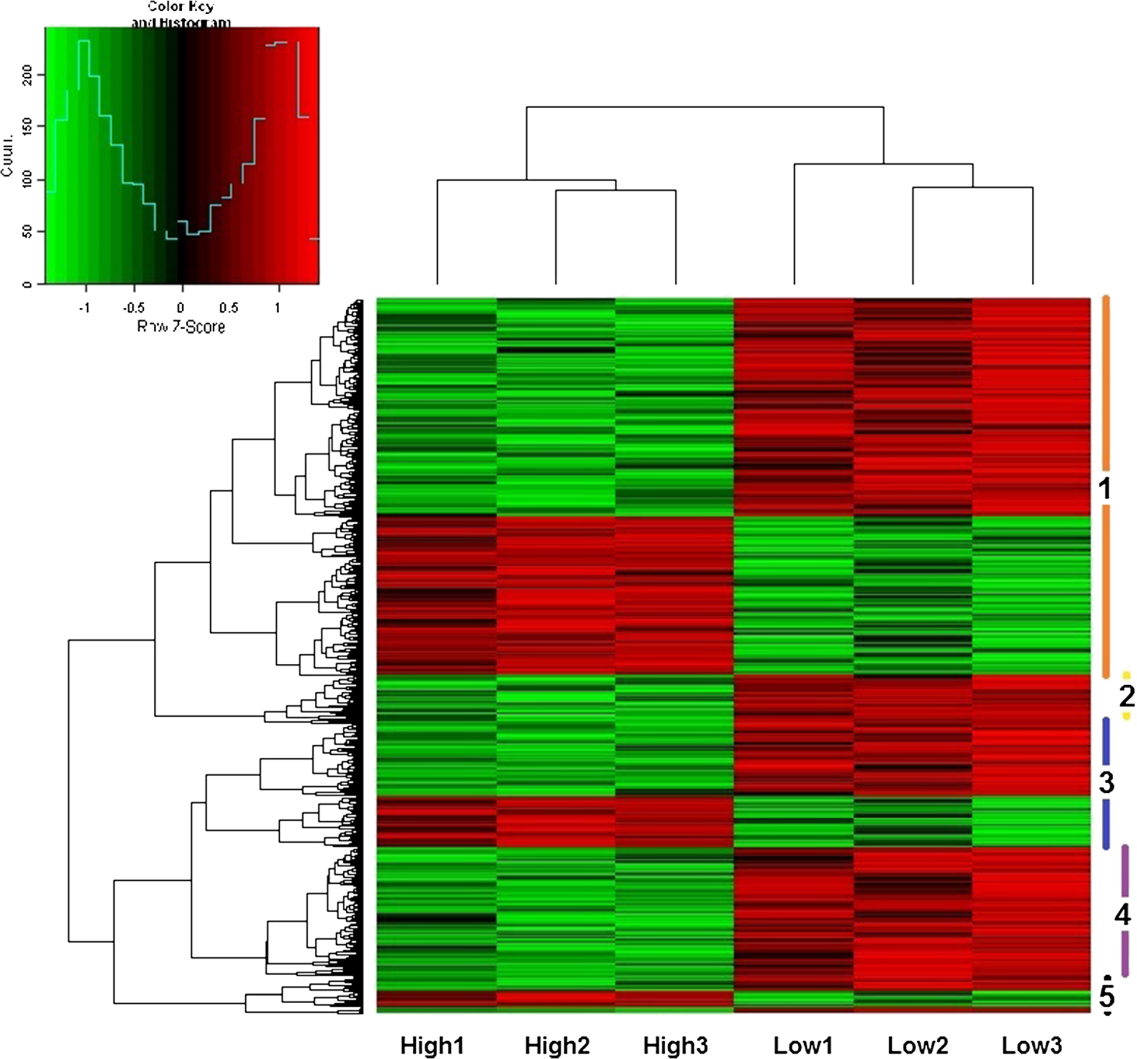
**Hierarchical clustering of all differentially expressed genes.** Colors indicate up- (red) or down- (green) regulation. Designation of clusters from 1 to 5 is displayed at the right.

To examine the co-expression of genes involved in photosynthesis, we used hierarchical clustering to classify the differentially expressed genes, based on their expression patterns, into 5 clusters (Figure 3). Two expression patterns (up- and down-regulated) were observed in most clusters, but the genes in cluster 2 and cluster 4 were all repressed (Figure 3). After SEA on each cluster, in cluster 3, we identified five significant GO terms, including polysaccharide metabolic process (GO:0005976), carbohydrate metabolic process (GO:0005975), hydrolase activity, acting on glycosyl bonds (GO:0016798), hydrolase activity, hydrolyzing O-glycosyl compounds (GO:0004553) and hydrolase activity (GO:0016787). Cluster 3 includes genes such as endoxyloglucan transferase, glycosyl hydrolase, beta-glucosidase, and xyloglucan endotransglucosylase/hydrolase protein, which encode hydrolases or transferases that act on xyloglucans in construction, modification, and degradation of plant cell walls. This result suggests that the expression of these genes is similarly regulated and these cell wall processes may be related to photosynthesis.

### Categories of differentially expressed genes involved in photosynthesis

The differentially expressed genes involved in photosynthesis were enriched in some groups including genes coordinating with organelles, and genes related to: the plant cell wall, response to stimulus, transport and redox. We also looked for organellar proteins and found 72 differentially expressed genes that encode proteins with known *Arabidopsis* homologs that localize in the chloroplast and/or mitochondrion (see Additional file 9). Gene expression ratios for the putative organellar proteins varied from 0.080- to 0.498- and 2.023- to 8.047-fold. A representation of the differentially expressed genes is shown in Additional file 10: Figure S2A. Of these, 80.95% of mitochondrion-located genes were down-regulated (Additional file 10: Figure S2B). This reveals that regulatory functions may be coordinated between nuclei and organelles in photosynthesis. We also identified 82 differentially expressed genes involved in cell wall functions (see Additional file 11). The SEA analysis in all different expression genes showed that cell wall macromolecule catabolic process (GO:0016998), cell wall macromolecule metabolic process (GO:0044036) and cell wall organization or biogenesis (GO:0071554) were significantly enriched GO terms (Table 2). However, the up- and down-regulated genes showed different related biological processes; most of the up-regulated genes related to plant cell wall growth and most of the down-regulated genes related to response for stimulus (Additional file 12: Figure S3, Additional file 11).

**Figure 4:**
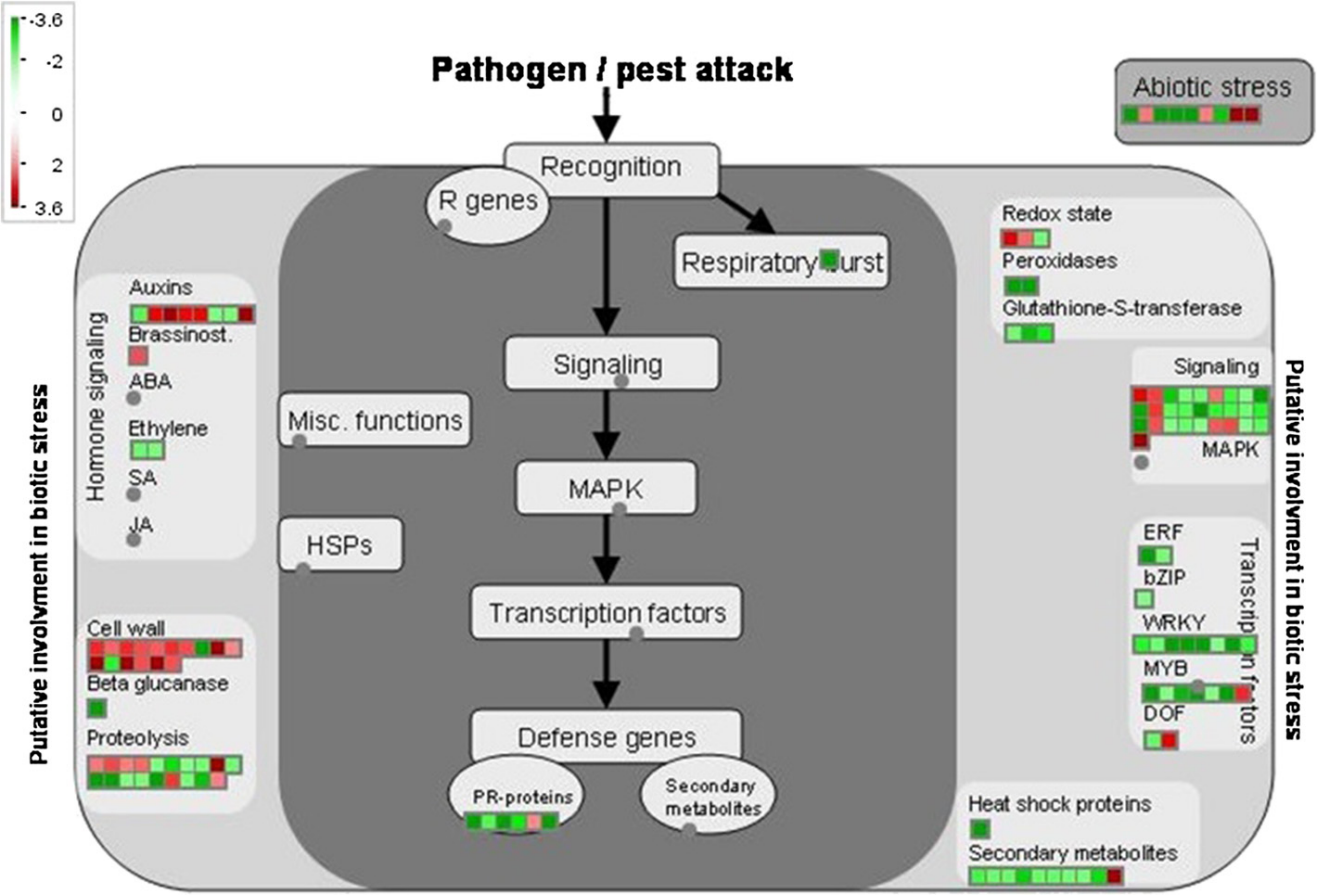
**Expression of genes involving in stress responses.** We mapped the differentially expressed genes identified as being involved in stress responses, and their expression ratios, onto different stress response pathways, as indicated. The ratios in the biological replicates were averaged, converted to a log2 scale, and imported into MapMan, which converts the data values to a false color scale. Transcripts called not present are shown as gray, transcripts that increased in plants with high photosynthetic efficiency are shown in red, and transcripts that decreased are green.

We also detected 83 genes affecting response to stimulus, and most of them (62.65%) were down-regulated (see Additional file 13). Guided by the *Arabidopsis* homologs, via MapMan (http://gabi.rzpd.de/projects/MapMan/) [36], we found that many are involved in biotic stress, such as hormone signaling (e.g. auxins, ethylene), transcription factors (e.g. WRKY, MYB), and abiotic stress (Figure 4). This suggested that response to stimulus was an important part of regulation of photosynthesis at the genome scale.

Transporters perform many important physiological functions, including the transport and distribution of photosynthetic assimilates. Hence, it was no surprise that genes related to transport were identified. We detected 45 differentially expressed genes (8.74% in all identified genes) related to transport (see Additional file 14). Gene families of these differentially expressed genes included ABC (ATP-Binding cassette), MFS (major facilitator superfamily protein), MIP (major intrinsic protein), OPT (oligopeptide transport), SLAH (S-type anion channel), PIN and other families. Most of the down-regulated genes encoded proteins predicted to locate in the membrane and conduct energy-related transport. This suggested that genes related to transport also affect photosynthetic rate. Photosynthesis has a high risk for producing photo-oxidative damage and generates redox intermediates with strong redox potentials (see Additional file 15). GO term enrichment analysis revealed that the biological process ‘oxidation reduction’ (GO:0055114, *P* = 0.0015) was also significantly enriched (Table 2). Four genes encoding glutathione transferase, one encoding glutathione and one encoding glutathione-dependent formaldehyde dehydrogenase were detected and all were slightly repressed in plants with high *Pn*, from 0.24- to 0.45-fold (see Additional file 15). Also, three NADH dehydrogenases, two NADH/Ubiquinone/plastoquinones (complex I) and two NADPH oxidases were repressed (see Additional file 15). This suggested that the regulation of redox plays a crucial role in photosynthesis.

### Polymorphic SNP loci associated with traits affecting photosynthesis

For functional examination of the differentially expressed genes identified in this study, we looked for association of SNPs with photosynthetic traits. We selected six candidate genes known to be involved in primary cell wall formation, hormone signaling and response to stress. We identified single nucleotide polymorphisms (SNPs) in each gene and carried out single SNP marker analysis with the photosynthesis parameters in our segregating population. (Additional file 16: Table S6). We found 158 common SNPs with a minor allele frequency greater than 0.10. Of these, 73 are in exon regions, 20 in 5′UTRs, 48 in introns, and 17 in the 3′UTR regions (Additional file 17: Table S7). We conducted 1738 (158 SNPs × 11 traits) single-marker analyses to account for linear regression by single factor ANOVA (see Additional file 18). We identified 34 associations that were significant at the threshold of *P* < 0.05 and multiple test corrections using the FDR (false discovery rate) method reduced this number to 20 at a significance threshold of FDR < 0.10 (Table 3). We identified significant associations with *Pn* (8 SNPs), *Cond* (6 SNPs), *Ci* (2 SNPs), *Trmmol* (3 SNPs) and tree height (1 SNPs) in the population (FDR < 0.10; Table 3). The 20 associations represented 9 SNP loci from the six candidate genes. Four candidate genes exhibited significant associations with at least three photosynthesis traits, suggesting pleiotropic effects or causal relationships among certain traits (Tables 1 and 3). Of these, association of *GASA3* (gibberellin-regulated protein) with *Cond* and *Pn* explained up to 12.6% and 8%, respectively, of the phenotypic variance we observed in the population*.* Most loci explained a small proportion of the phenotypic variance ranging from 2.3% to 8.0%. These small SNP effects are in accordance with polygenic quantitative models of complex traits [37], and support the opinion that photosynthesis should be examined on a whole-plant level.

**Table 3:** Significant SNP marker-trait associations tested in the population (n = 300) after a correction for multiple testing [false discovery rate FDR ≤ 0.10]

**Trait**	**Gene symbol**	**Locus**	**Missense mutation**	***P*-value**	**Phenotypic variation (%)**	**FDR**	**Frequency (%)**	
*Pn*	XET 5′UTR	XET-SNP2		< 0.001	5.4	0.0003	0.48	(G)
	XET 5′UTR	XET-SNP3		< 0.001	7.2	0.0006	0.41	(T)
	XET Exon1	XET-SNP5	Leu to Pro	< 0.001	5.2	0.0012	0.41	(C)
	XET Exon1	XET-SNP6	Ala to Pro	< 0.001	5.4	0.0009	0.38	(C)
	Dabb Exon2	Dabb-SNP2	Ala to Val	0.001	4.8	0.0019	0.45	(T)
	GASA 3′UTR	GASA-SNP3		< 0.001	8.0	< 0.0001	0.46	(C)
	SAUR Exon	SAUR-SNP1	Glu to Gln	0.008	2.3	0.0158	0.26	(G)
	CGSS Exon1	CGSS-SNP1	Met* to Val	< 0.001	7.0	< 0.0001	0.15	(G)
*Cond*	XET 5′UTR	XET-SNP3		< 0.001	7.9	< 0.0001	0.41	(T)
	XET Exon1	XET-SNP6	Ala to Pro	< 0.001	5.9	0.0005	0.38	(C)
	Dabb Exon2	Dabb-SNP2	Ala to Val	< 0.001	7.0	< 0.0001	0.45	(T)
	GASA 3′UTR	GASA-SNP3		< 0.001	12.6	< 0.0001	0.46	(C)
	SAUR Exon	SAUR-SNP1	Glu to Gln	< 0.001	5.8	< 0.0001	0.26	(G)
	CGSS Exon1	CGSS-SNP1	Met* to Val	< 0.001	6.8	< 0.0001	0.15	(G)
*Ci*	XET 5′UTR	XET-SNP3		0.003	3.8	0.0073	0.41	(T)
	SAUR Exon	SAUR-SNP1	Glu to Gln	0.007	2.4	0.0149	0.26	(G)
*Trmmol*	XET 5′UTR	XET-SNP3		0.002	4.3	0.0040	0.41	(T)
	Dabb Exon2	Dabb-SNP2	Ala to Val	0.004	3.6	0.0100	0.45	(T)
	CGSS Exon1	CGSS-SNP1	Met* to Val	0.008	2.3	0.0158	0.15	(G)
*H*	PI 3′UTR	PI-SNP1		0.005	2.6	0.0651	0.38	(T)

*Pn*, photosynthetic rate; *Cond*, conductance to H_2_O; *Ci*, intercellular CO_2_ concentration; *Trmmol*, transpiration rate; *H*, tree height; Frequency, Allele frequency of either the derived or minor allele. Single nucleotide polymorphism (SNP) alleles corresponding to the frequency listed are given in parentheses.

### Regulatory network of nuclear genes involved in photosynthesis

**Figure 5:**
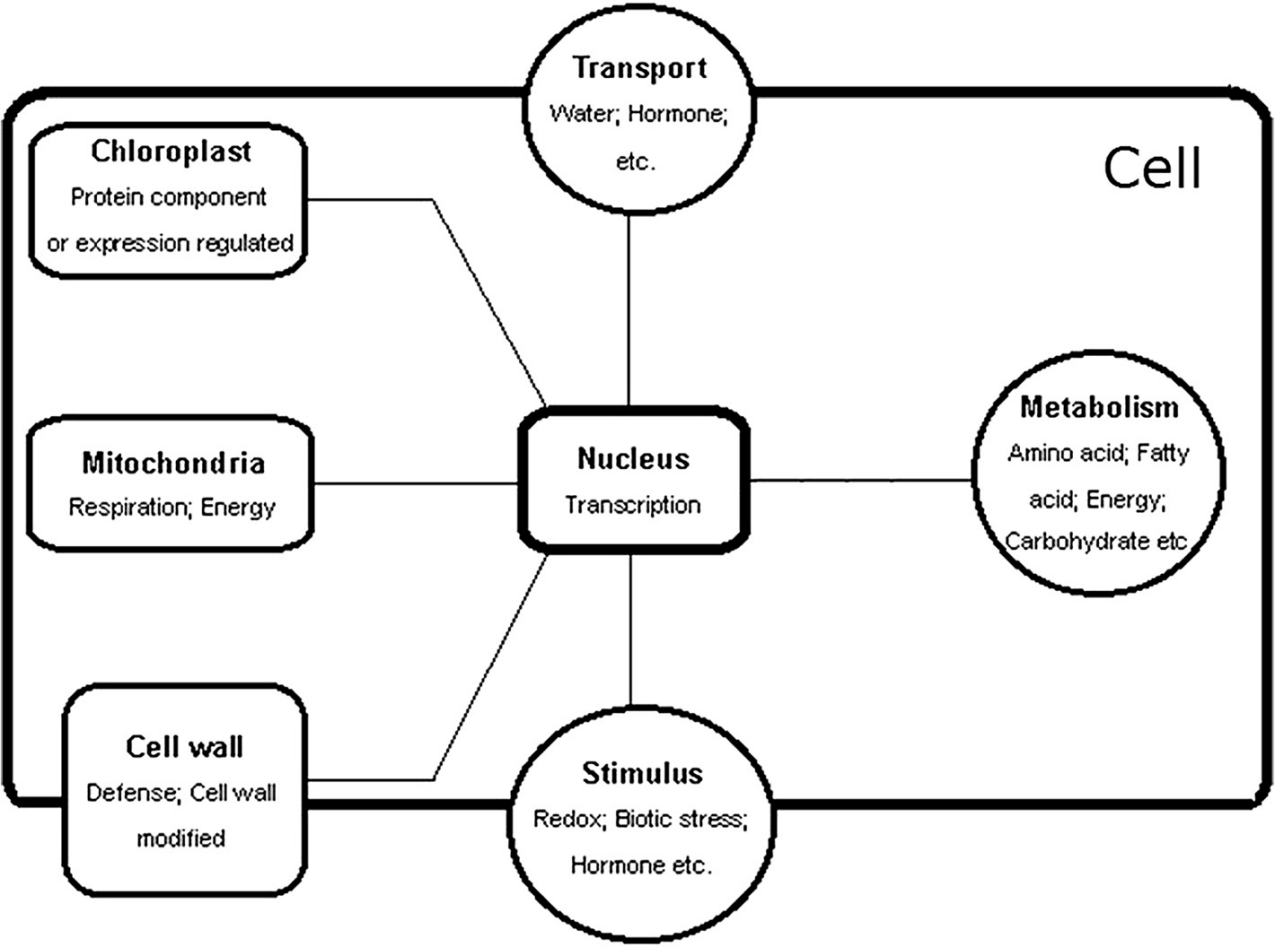
**A diagram of the regulation of photosynthesis by nuclear genes.** The diagram depicts the main cell components (in squares) and processes (in circles) that were identified in this study. Annotation reflects the function of the identified genes. This figure is a simple draft, in which the relationship between components and processes is not considered.

After we identified differentially expressed genes, we constructed a gene network (Figure 5) to reflect the processes and organelles that the nuclear genome controls in photosynthesis. The main cell components included chloroplasts (Fig. S2A), mitochondria (Fig. S2B) and the cell wall (Fig. S3). It seemed likely that the nucleus affects the structure of chloroplasts and the regulation of chloroplast gene expression, the coordination of oxidative phosphorylation in mitochondria and modification of the cell wall in defense or growth. According to the annotation, regulation of the nuclear genome involving photosynthesis mainly involved: transport (e.g., ABC, MFS), metabolism (e.g., glucanase, beta-D-xylosidase, NADH dehydrogenase) and response to stimulus (e.g., WRKY, chitinase). Of these, metabolism involved a number of physiological and biochemical processes including carbohydrates, amino acids, lipids, fatty acids and energy. This suggested that the regulation of the nuclear genome concentrated on various biological processes (e.g., carbohydrate synthesis, environmental adaptation, transport efficiencies of water, assimilates and nutrients) rather than just affecting chloroplast-related processes (e.g., the primary events of light harvesting and carbon fixation) [7] although coordination between the nucleus and chloroplast was crucial in maintaining normal photosynthesis.

## Discussion

Our study identified 515 nuclear genes involved in photosynthesis in *Populus* under field conditions. Based on our analysis of these differentially expressed genes, we constructed a gene network (Figure 5), which revealed the role of nuclear genes involved in photosynthesis under field conditions in *Populus*. The major processes identified by these genes include response to stimulus, metabolism, and transport. This study provides new genome-scale strategies for the discovery of potential candidate genes affecting photosynthesis in *Populus*, and also for identification of gene functions involved in the regulation of photosynthesis.

### Genes coordinating with organelles

Enzymatic and photosynthetic complexes within chloroplasts contain subunits encoded by the nuclear and plastid genomes [12], and chloroplasts and mitochondria interact with the nucleus. Hence, we compared our study with previous studies to find genes involved in coordinating nuclear functions with organelles (chloroplast and/or mitochondrion) at the genome scale under field conditions. We detected 72 differentially expressed genes encoding putative organellar proteins (Additional file 10: Figure S2A). The largest groups of differentially expressed genes included genes involved in metabolism (18.31%, e.g. NADP^+^-isocitrate dehydrogenase, sulfotransferase 2A), photosynthesis (23.94%, e.g. chlorophyllase, PsbP-like protein 1) and genes with unknown function (19.72%) (Additional file 9). Of these, 80.95% of mitochondrion-located genes were down-regulated (Additional file 10: Figure S2B) including acyl activating enzyme (*FC* = 0.106), enoyl-CoA hydratase/isomerase (*FC* = 0.432), and cytochrome P450 (*FC* = 0.120). Most of these genes play particularly important roles in different metabolic processes, including energy metabolism or downstream molecular functions affecting metabolic processes. For example, enoyl-CoA hydratase/isomerase indicates an effect on the β-oxidation pathway, which might change carbon flux (in the form of acetyl-CoA) entering the tricarboxylic acid cycle [38]. The large cytochrome P450 of plant metabolic enzymes catalyze a huge variety of oxidation reactions in microbes and higher organisms [39]. Mitochondrial oxidative metabolism modulates the coordination between different components of photosynthetic carbon assimilation in chloroplasts, such as generation and use of assimilatory power (ATP and NADPH), induction of photosynthesis, maintenance of metabolites and the light activation of Calvin cycle enzymes [40,41]. The above considerations indicate that the mitochondrion plays an important role in photosynthesis.

As expected, we detected nuclear genes involved construction or development of chloroplasts (e.g., senescence-inducible chloroplast stay-green protein, PsbP-like protein) (see Additional file 9). For example, the gene encoding ATP-dependent Clp protease regulatory subunit, an essential housekeeping enzyme in chloroplasts [42], was down-regulated by 0.45-fold. PsbP-like protein is an extrinsic subunit of eukaryotic photosystem II (PSII) and participates in the normal function of photosynthetic water oxidation; the up-regulation of this gene (2.14-fold) revealed that nuclear genes control photosynthesis by affecting the structure or development of chloroplasts.

We found that co-ordination between the nucleus and chloroplast was a crucial but small part of the nuclear regulation of photosynthesis. We did not identify significant GO terms involving chloroplast-related processes. After comparing our results to a previous study, we identified just 20 genes (e.g., chloroplast precursor, potassium channel KAT3, and phosphate-responsive 1 family protein) in the analysis of 101 nuclear transcriptomes, which revealed 23 distinct regulons and their relationship in coordination of nuclear and plastid gene expression [43] (Additional file 19: Table S8). However, genes related to other complex biological processes were identified in our study. Hence, we consider the regulation of photosynthesis in trees should be viewed on a whole-plant scale.

### The role of the cell wall in photosynthesis

We identified 82 differentially expressed genes involved in cell wall functions (Additional file 11). Although a similar number of genes were up- (39) and down-regulated (43) (Additional file 12: Figure S3), the constitution of related biological processes clearly differed. Most of the up-regulated genes were related to plant cell wall growth, such as expansins (*FC* = 2.009 to 5.536) and xyloglucan endotransglycosylase (*FC* = 2.192 to 11.412). Conversely, most of the down-regulated genes were related to response for stimulus, such as chitinase (*FC* = 0.148 to 0.422) and pathogenesis-related protein (*FC* = 0.046). This revealed that photosynthesis may be positively correlated with growth and negatively correlated with stress.

As expected, we detected genes related to carbohydrate active enzymes (CAZYmes), which function in the formation and modification of the carbohydrate matrix of wood cell walls, including genes encoding cellulose synthase-like (CSLs), pectin methyl esterases (PMEs), xyloglucan endo-transglucosylases and hydrolases (XETs and XEHs), and expansins [44]. These proteins play crucial roles in assimilating the products of photosynthesis into sugars and starch, in synthesizing cell wall biopolymers and in creating various glycosylated compounds [45]. Most of the carbohydrate-related genes (75%) were slightly up-regulated, by 2.01- to 11.41-fold, indicating that high photosynthetic rate was related to carbohydrate activity. Of these, CSLs (*FC* = 2.69- to 2.70-fold), which are good candidates for the synthases forming the β-D-glycan backbone of hemicelluloses such as xyloglucan, xylan, mannan and other β-D-glycans in the cell wall, were up-regulated [46]. PMEs, which were among the up and down-regulated genes, have major roles in pectin remodeling [47]. These genes influence cell wall remodeling, which may largely contribute to internal carbon recycling and thus ultimately affect photosynthesis [48].

XET specifically cuts the backbone of xyloglucan and re-forms a glycosidic bond with the free end of another xyloglucan chain [49]. We found that the gene encoding XET was up-regulated by up to 11.41-fold in the poplars with higher photosynthetic efficiency. As the most abundant hemicellulose in the primary cell wall in many dicotyledonous plants, xyloglucan has a backbone of β1 → 4-linked glucose residues. This may be affected by sugar signaling pathways, as sucrose is the major photosynthetic product and affects growth and metabolism [50]. This also supports the present result that four SNP markers from a xyloglucan endotransglycosylase precursor (XET16A) showed a significant association with four photosynthetic characteristics (Table 3). Of these, a significant SNP (XET-SNP3) detected in the 5′ UTR may affect many post-transcriptional regulatory pathways. The T-C mutation in XET-SNP5 causes a leucine to proline amino acid substitution and the G-C mutation at XET-SNP6 causes an alanine to proline substitution. These findings suggest that XET may function downstream of photosynthesis and play an important role in the balance of glucose. In *Populus*, at least 16 XTH genes, likely encoding XETs, were expressed in developing xylem, indicating that the amount of nascent xyloglucan relative to XET was an important determinant of whether XET strengthens or loosens the cell wall [51]. This supports the idea that this gene is involved in photosynthesis in *Populus*.

We found that of the genes down-regulated in plants with high photosynthetic efficiency were related to stress responses, such as PI (proteinase inhibitor), chitinase [52] and pathogenesis-related protein [53], and the fold change of these genes was as low as 0.03 (PR-6 proteinase inhibitor). Of these, PI-SNP1, a noncoding marker within a gene from the PR-6 proteinase inhibitor family constituting a sub-class of serine PIs with characteristics of potato/tomato type I PIs [54], was the only single-marker association with a growth trait (*H*) in single marker analysis (Table 3). Plants stringently govern PI activity to achieve cellular homeostasis. We suppose that PI activity relates to tree height through its effect on the water-stress response. Current hypotheses for tree height limitation focus on increasing water transport constraints in taller trees and the resulting reductions in leaf photosynthesis [55]. Also, PIs were up-regulated under water stress both in soybean [56] and maize [57].

### Photosynthesis and hormone

Plant hormones, a group of chemically diverse small molecules, direct processes ranging from growth and development to biotic and abiotic stress responses. In this study, the stimulus response category related to hormone responses included SAUR (small auxin up-regulated RNA), AUX (auxin response gene family), GASA (gibberellin-regulated protein), GID (gibberellin receptor) and some genes responding to other hormones (Additional file 13). The genes showed clear differences between up- and down-regulated gene sets. For instance, all the GIDs were down-regulated and the vast majority of SAUR, AUX and GASA genes, which function in different processes throughout plant growth and development [58], were up-regulated. For example, SAURs function as primary auxin response genes hypothesized to be involved in auxin signaling pathways. They were up-regulated by 2.32- to 3.98-fold. Genes encoding auxin efflux carrier family proteins were also up-regulated, suggesting that photosynthetic activity might increase auxin content to affect development [59]. The effect of GA on photosynthesis has been controversial [60-63]. In our study, a gene encoding GASA was up-regulated by 4.03-fold. The SNP marker in *GASA* showed a significant association with two photosynthetic traits (Table 3). Phenotypic variance for *Cond* and *Pn* explained by the GASA-SNP3 marker was up to 12.6% and 8.0%, respectively. Our result supports the idea that photosynthesis was at least indirectly GA-responsive, an idea further supported by a study in which GA3 treatment increased photosynthetic activity [64].

### Photosynthesis and stress

Environmental factors limit photosynthesis, so plants rarely realize their maximum photosynthetic potential under field conditions [7]. Hence, it was not surprising that we found genes related to stress in this study. Stress-related genes likely play roles in protecting photosynthetic functions, metabolizing the factors that inhibit photosynthesis [25,29] and maintaining cell homeostasis. For example, we found that peroxiredoxin was down-regulated by 0.02-fold in the poplars with high photosynthetic efficiency (Additional file 15). It encodes peroxiredoxin Q, which decomposes peroxides and plays a role in the protection of the photosynthetic apparatus [65]. Also glutathione-transferase (GST) (down-regulated by 0.24- to 0.45-fold) is induced by diverse environmental stimuli, with increased GST levels used to maintain cell redox homeostasis and protect organisms from oxidative stress, which can seriously affect leaf photosynthetic machinery [66]. Also, we found that nuclear genes affect endogenous and environmental stimuli and regulate key proteins responding to stress. We detected 14 WRKY transcription factors, important functional components for plant stress responses, as being down-regulated. WRKYs relate to tolerance to wounding, heat, cold, NaCl, sugar, drought, oxidative stress and others [67] (see Additional file 20).

SEA analysis showed an enrichment of terms involving chitinase, such as chitin catabolic process (GO:0006032), chitin metabolic process (GO:0006030), chitinase activity (GO:0004568) and chitin binding (GO:0008061) (Table 2). Chitinase protects plants against fungal pathogens by degrading chitin, a major component of the cell walls of many fungi (see Additional file 11). Fragments from chitin and chitosan have elicitor activities leading to a variety of defense responses in host plants in response to microbial infection [68], including induction of pathogen-related proteins and PI, which were also identified in this study.

Most genes involved in stress response were down-regulated, such as PI, chitinase, peroxidase, heat shock protein [69] and pathogenesis-related protein. For example, all six genes encoding peroxidase, a plant-specific oxidoreductase affecting responses to environmental stimuli [70], were repressed, down to 0.01-fold. Also, peroxiredoxins play important roles in plant development, environmental adaptation, and disease resistance [71], and this gene was repressed too. This result could be considered from two aspects. First, low photosynthetic rate led to less production of sugar and energy, resulting in a decline in plant adaptability [72]. Second, plants with good environmental adaptability could grow and thus had a high photosynthetic rate. All the above results suggest that nuclear regulation of the response for stress is an important part of photosynthesis. This suggests that improving plant stress tolerance may enhance photosynthetic efficiency in field conditions.

### Photosynthesis and metabolism

The relationship between metabolism and photosynthesis is complex, involving photosynthate production (e.g., carbohydrates, proteins, amino acids etc.), energy and nutrition. Cell wall carbohydrate metabolic processes were highlighted in this study. This suggests that nuclear genes may affect photosynthesis by regulating polysaccharides in the cell wall for a possible source of sugar. For example, the identified cell wall-bound β-glucosidase was repressed (*FC* = 0.07-fold) in high photosynthetic rate pools (see Additional file 11). It breaks down polysaccharides to soluble sugars when photosynthesis declines. Also, reduced photosynthesis results in enhancement of β-glucosidase activity [73].

Energy metabolism was also detected in our study. The complex network of plant energy metabolism involves photosynthesis, photorespiration, nitrogen and sulfur reduction, and other molecules [24]. Our study identified genes affecting ATP/NADPH, such as ADP-ribose pyrophosphatase, NADH dehydrogenase, NADH/Ubiquinone/plastoquinone (complex I), and NADPH oxidase (see Additional file 15). Most of these genes also play roles in redox. The gene encoding ADP-ribose pyrophosphatase (up-regulated by 2.40-fold), conferred enhanced tolerance to oxidative stress in Arabidopsis plants, resulting from maintenance of NAD + and ATP levels by nucleotide recycling from free ADP-ribose molecules [74]. Also, plant extracellular ATP results in the activation of plasma membrane NADPH oxidases, causing the production of reactive oxygen species [75]. In addition, NAD(P)H dehydrogenases are related to respiratory complex I, and mediate photosystem I cyclic electron flow. Hence, we consider that energy metabolism is an aspect of the nuclear regulation of photosynthesis.

### Photosynthesis and transport proteins

Transporters perform many important physiological functions, including the transport and distribution of photosynthetic assimilates. However, our study did not detect the well-known sucrose transporters, such as sucrose uptake transporters (SUTs) [76]. This result may be caused by annotation of differentially expressed genes, which should be improved in future work. For example, MFS, a typical representative of secondary transporters and the largest known superfamily of secondary carriers found in the biosphere [77], was detected in our study (*FC* = 0.32 to 0.46). Plant sucrose transporters belong to this superfamily and can mediate carbohydrate transport across cell membranes [78]. ABC transporters, arguably the most important family of ATP-driving transporters in biology, have been implicated in a broad range of processes, including polar auxin transport, disease resistance, and stomatal function [79]. In a study of *Butyrivibrio proteoclasticus*, 12 ABC transport system substrate-binding proteins were predicted to function as carbohydrate transporters [80].

Transport proteins are embedded in the membrane to enable the passage of metabolites and thereby connect metabolic networks beyond organelle boundaries [81]. Transporters may affect photosynthetic rate via metabolite transport, hormone signaling and sustaining cell homeostasis. The transport proteins we detected include phosphate transporter (*FC* = 0.12), PIN1-like auxin transport protein (*FC* = 5.37) and potassium channel KAT3 (*FC* = 0.31) (see Additional file 14). Transport proteins are important for achieving high rates of photosynthetic carbon assimilation [10,82]. Also, in hormone signaling, PIN transporters (up-regulated by 5.37-, 2.97-fold) play important roles in directional auxin distribution within tissues [83]. Recent studies have suggested that the HAK family transporters (down-regulated by 0.21-fold) are potentially involved in K^+^ homeostasis and osmotic regulation in plants [84]. Also, transport plays an important role in maintaining cellular homeostasis for adaptation to environmental stress [85]. For example, the OPT family consists of electrochemical potential–driven transporters [86]. AtOPT6 can mediate uptake of glutathione derivatives and metal complexes, indicating that it may also be involved in redox homeostasis [87].

## Conclusions

Here we report the genome-wide identification and characterization of nuclear genes involved in photosynthesis. This study detected 515 differentially expressed genes in a segregating linkage population of poplar (*FC* ≥ 2 or *FC ≤* 0.5, *P* < 0.05). Our study suggests that improving photosynthetic efficiency should consider different regulatory levels, rather than just the primary reactions of carbon assimilation under field conditions. Our study also reflects a general picture of regulation of photosynthesis from the nucleus in a normal environment. This study will be a part of further analysis using systems biology, a multidisciplinary science that uses large data sets to generate hypotheses about a dynamic system and has been effectively used to study signaling networks [18]. Identification and characterization of nuclear genes involved in photosynthesis in *Populus* provides new insights in the molecular mechanisms that regulate photosynthesis in field and information that may enable efforts to improve photosynthetic efficiency in trees.

## Methods

### Plant materials and growth condition

A total of 1200 F_1_ progeny, which were randomly selected from the F_1_ hybrid population of 5000, were used in this study. The population was established by controlled crossing between two elite poplar parents, the female hybrid clone “YX01” (*P. alba* × *P. glandulossa*) and the male clone “LM50” (*P. tomentosa*), two species of the section *Populus*. The progeny were grown in Xiao Tangshan horticulture fields of Beijing Forestry University, Beijing, China (40°2′N, 115°50′E), using a randomized complete block design with three replications in 2008.

### Measurement of photosynthetic characteristics and quantitative traits

Photosynthetic characteristics were measured from fully expanded leaves using the LI-6400 portable photosynthesis system (Li-Cor Inc., Lincoln, NE, USA) at 9:00–11:00 on sunny days from July 20th to August 8th, 2010. Growing conditions during the experimental time window are shown in Additional file 21: Table S9. Three functional leaves, the fourth to sixth counting from the individual stem head of all of the individuals, were measured with three replications for each leaf. Values of photosynthetic parameters, such as *Pn*, *Cond* and *Ci*, were automatically recorded when *Pn* showed no systematic decrease or increase (± 0.1). Besides, seven quantitative traits including *H, D* (1.3 m above the ground), *V, MFA* and holocellulose, α-cellulose, and lignin contents were measured in all three replicates of the 1,200 clones in the hybrid population described by Du et al. (2013) [88]. Broad-sense heritability was calculated as in Singh et al. (1993) [89].

### Bulk segregant analysis and RNA extraction

According to the average value of each individual for 1200 progeny, fifty individuals with values distributed in the extreme area were measured again to ensure the stable phenotypic variance in photosynthetic characteristics. Among them, fifteen individuals with high (*Pn* > 21.000 μmol•m^−2^•s^−1^) and fifteen individuals of low photosynthetic rate (*Pn* < 11.000 μmol•m^−2^•s^−1^) were selected to construct gene pools by BSA according to Michelmore et al. (1991) [35], to produce two pooled samples with genetically dissimilar photosynthetic rates. All leaf samples were immediately placed into liquid nitrogen in the field and stored at −80°C until used for isolating RNA. Samples were segregated into two groups (high and low) and five individuals were pooled, producing three biological repeats for each group. These high *Pn* groups were named High1, High2, High3. And low *Pn* groups were named Low1, Low2, Low3. A simple *t*-test using the Statistic Package for Social Science (SPSS) among these pools showed a significant difference between high and low photosynthetic rate groups (Additional file 22: Table S10) (*P* ≤ 0.01). Total RNA was extracted using the Plant Qiagen RNAeasy kit (Qiagen China, Shanghai) according to the manufacturer’s instructions. RNA extractions of 5 individuals were mixed in each replicate to equal quantities (2 mg) for later microarray analysis. All the RNA samples (individual and mixed) were examined by Agilent 2100 Bioanalyzer (Agilent Technologies, USA) according to the manufacturer’s instructions, and no evidence of degradation was noted. The RNA samples were reverse transcribed into cDNA using the SuperScript First-Strand Synthesis system and the supplied polythymine primers (Invitrogen). In total, six gene pools were constructed, representing samples with three high and three low photosynthetic rates, respectively.

### Microarray experiments

Array hybridization and washes were performed using the GeneChip Hybridization, Wash and Stain Kit (cat. #900720; Affymetrix, Santa Clara, CA) in a Hybridization Oven 645 (cat. #00-0331-220 V; Affymetrix) and Fluidics Station 450 (cat. #00-0079; Affymetrix) following the manufacturer’s instructions. The array contains 61,251 poplar probe sets (including seven rRNA probe sets), 12 poplar control probe sets, and 62 reporter probe sets. Slides were scanned by GeneChip Scanner 3000 (cat. #00-00212; Affymetrix) and Command Console Software 3.1 (Affymetrix) with default settings. Raw data were normalized via the MAS 5.0 algorithm, Gene Spring Software 11.0 (Agilent Technologies). Due to the high technical consistency and reliability of microarrays, technical replications of hybridizations were not performed. cRNA labeling and hybridization to the Poplar Genome Array were performed by the ShanghaiBio Corporation in China. The expression data of all samples were uploaded to the Gene Expression Omnibus (http://www.ncbi.nlm.nih.gov/geo/) with accession number GSE47105.

### Quantitative real-time PCR validation of expression profiles

Quantitative PCR was performed on a DNA Engine Opticon 2 machine (MJ Research) using the LightCycler-FastStar DNA master SYBR Green I kit (Roche). The PCR program was described in Zhang et al. [90]. The melting curve was used to check the specificity of the amplified fragments. All reactions were carried out in triplicate for biological repeats, and the real-time data were analyzed using the Opticon Monitor Analysis Software 3.1 tool. Specific primer sets were designed for each gene using Primer Express 3.0 software (Applied Biosystems) (Additional file 6: Table S3). The efficiency of the primer sets was calculated by performing real-time PCR on several dilutions of first-strand cDNAs. Efficiencies of the different primer sets were similar. The specificity of each primer set was checked by sequencing PCR products. The results obtained for the different samples were standardized to the levels of *Actin*.

### Biological interpretation and cluster analysis

Gene annotations were obtained from GenBank (http://www.ncbi.nlm.nih.gov/genbank/), JGI (http://www.phytozome.net/poplar), PopGenie v2.0 (http://popgenie.org/flashbulktools) and TAIR (http://www.arabidopsis.org/). CateGOrizer (http://www.animalgenome.org/tools/catego/) was used for GO Term classification. We categorized differentially expressed genes by their Gene Ontology class using SEA, which was performed on AgriGO (http://bioinfo.cau.edu.cn/agriGO/) to identify the significantly enriched biological processes, cellular components and molecular functions. Complete-linkage hierarchical clustering was done using SAS for the expression pattern of specific genes at the SBS server of Ebioservice (http://www.ebioservice.com/).

### DNA extraction, SNP discovery, genotyping and single-marker analysis

Total genomic DNA was isolated from young leaves using a DNeasy Plant Mini Kit (Qiagen China, Shanghai) following the manufacturer’s protocol. To determine the SNP loci segregating in the F_1_ population of “YX01” × “LM50”, the genomic sequences of six candidate genes, i.e. *XET*, *Dabb*, *GASA*, *SAUR*, *CGSS*, and *PI*, were identified from the parents. The primer sets of these candidate genes used for the amplification were designed based on the *P. trichocarpa* genome sequence. In total, 96 sequences of candidate genes (6 genes × 8 *YX01*, 6 genes × 8 *LM50*) were obtained by sequencing the female hybrid clone “YX01” (*P. alba* × *P. glandulossa*) and the male clone “LM50” (*P. tomentosa*). After re-sequencing, Sequencher v.4.0 and BioEdit were used for sequence alignment and manual editing was used to confirm sequence quality and remove primer sequences. Diploid sequences were disambiguated into haplotypes using Phase v. 2.1 using 10,000 iterations of the Bayesian MCMC chain (Stephens and Scheet 2005). Subsequently, SNP discovery was performed with MEGA 4.0. The sequence data from this article has been deposited in the GenBank Data Library under the accession Nos. JX431909-JX431932. In total, 300 individuals of 1200 progeny were randomly selected for genotyping and single-marker analysis. 158 common SNPs (Minor Allele Frequency > 0.10) were genotyped in this association population by using locked nucleic acid technology [91]. Single-marker analysis based on the SNP markers and 11 traits was performed in the F_1_ hybrid population using single factor ANOVA. Percent phenotypic variance explained by the most significant marker was calculated, and the positive FDR with 10^4^ permutations was used to perform a correction for multiple testing in QVALUE (Storey and Tibshirani 2003) [92].

## Competing interests

The authors declared that they have no competing interests.

## Authors’ contributions

Conceived and designed the experiments: DZ. Performed the experiments: BW DZ QD XY. Analyzed the data: BW DZ. Contributed reagents/materials/analysis tools: BW DZ. Wrote the paper: BW DZ QD XY. All authors read and approved the final manuscript.

## Supplementary Material

Additional file 1: Figure S1 Frequency distribution of photosynthetic characteristics in the 1200 progeny. *Pn*, photosynthetic rate; *Cond*, conductance to H_2_O; *Ci*, intercellular CO_2_ concentration; *Trmmol*, transpiration rate. Click here for file

Additional file 2: Table S1 Phenotypic data obtained from hybrids. Click here for file

Additional file 3: Table S2 Mean squares of ANOVA and broad heritabilities for the traits. Click here for file

Additional file 4 **A list of 162 up-regulated genes in plants with high *Pn *** **compared to plants with low *Pn.***
Click here for file

Additional file 5 **A list of 353 down-regulated genes in plants with high *Pn *** **compared to plants with low *Pn.***
Click here for file

Additional file 6: Table S3 Primers used for real-time PCR analysis. Click here for file

Additional file 7: Table S4 The result of *t*-test analysis. Click here for file

Additional file 8: Table S5 Gene ontology enrichment analysis for the differentially expressed genes. Click here for file

Additional file 9 Annotation of genes coordinating with organelles. Click here for file

Additional file 10: Figure S2 Differentially expressed genes coordinating with organelles. Colors indicate up- (red) or down- (green) regulation. High1, High2, and High3 represent the pools of high photosynthetic rate plants and Low1, Low2, and Low3 represent the pools of low photosynthetic rate plants. In total, 72 differentially expressed genes related to organelles (chloroplast and/or mitochondrion) were identified. Of these, 27 genes were up-regulated and 45 were down-regulated in high photosynthetic rate gene pools. (A) Hierarchical clustering of differentially expressed genes coordinating with organelles. (B) Hierarchical clustering of differentially expressed genes involved with mitochondrial processes. Click here for file

Additional file 11 Annotation of genes related to the plant cell wall. Click here for file

Additional file 12: Figure S3 Differentially expressed genes related to the cell wall. In total, 82 differentially expressed genes related to the plant cell wall were identified. Of these, 39 genes were up-regulated and 43 were down-regulated. (A) Hierarchical clustering of cell wall related genes. The number of up- and down-regulated genes is approximate. (B) Functional classification of cell wall related genes. There were clear differences between up- and down-regulated genes. 46.15% of the genes were grouped into metabolism and 38.46% genes related to response for stimulus. Click here for file

Additional file 13 Annotation of genes affecting response to stimulus. Click here for file

Additional file 14 Annotation of genes involved in transport. Click here for file

Additional file 15 Annotation of genes related to redox. Click here for file

Additional file 16: Table S6 The annotation of candidate genes for SNP analysis. Click here for file

Additional file 17: Table S7 Number and distribution of SNPs detected in this study. Click here for file

Additional file 18 Experimental data of 1738 (158 SNPs × 11 traits) single-marker analyses to account for linear regression by single factor ANOVA (n = 300). Click here for file

Additional file 19: Table S8 Comparison with 1590 genes contained in the 23 regulons. Click here for file

Additional file 20 A list of transcription factors. Click here for file

Additional file 21: Table S9 Growing conditions in Xiao Tangshan horticulture fields. Click here for file

Additional file 22: Table S10 *T*-test analysis of BSA pools (n = 30). Click here for file
