# Supplementary figures and images for: Identification and characterization of nuclear genes involved in photosynthesis in Populus
Source: BMC Plant Biol. 2014 Mar 27;14:81. doi: 10.1186/1471-2229-14-81 (PMC3986721; doi:10.1186/1471-2229-14-81)

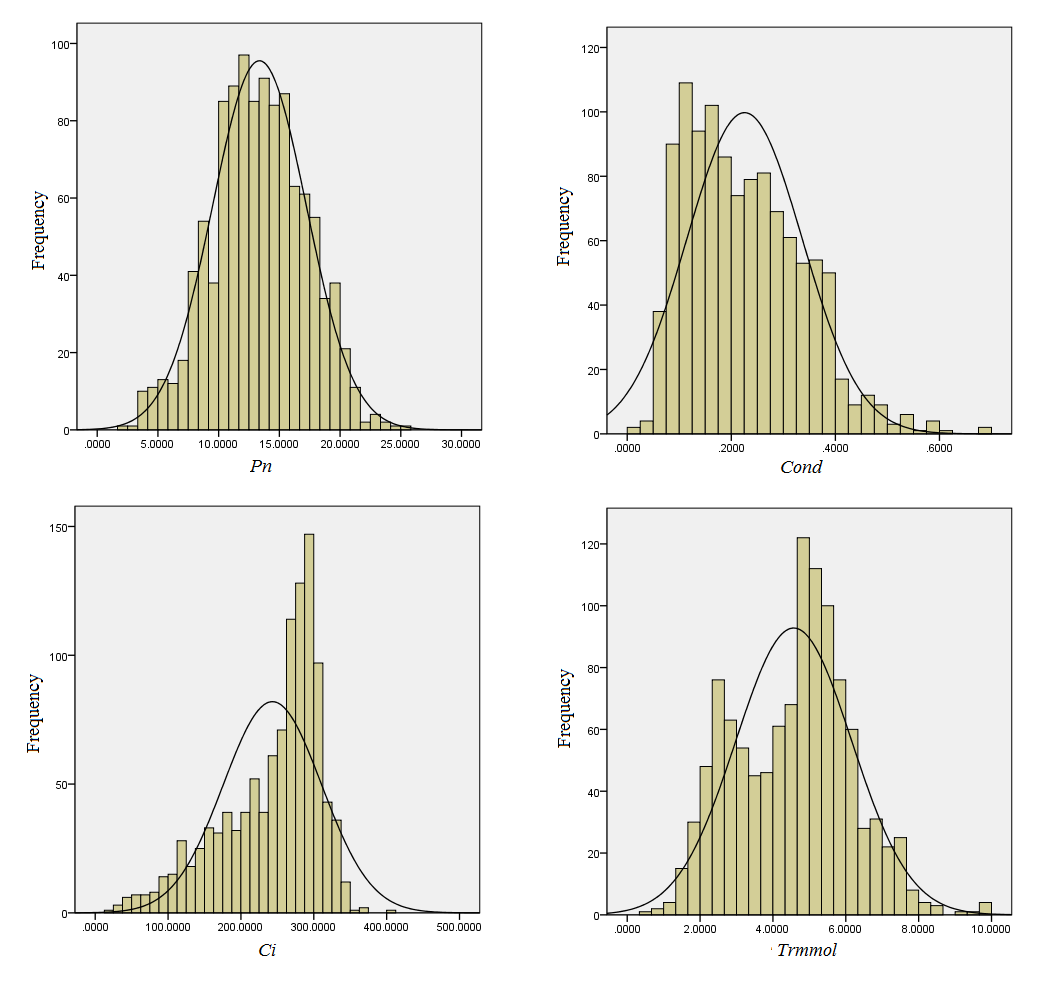

Supplement: Additional file 1: Figure S1 — Frequency distribution of photosynthetic characteristics in the 1200 progeny. Pn, photosynthetic rate; Cond, conductance to H2O; Ci, intercellular CO2 concentration; Trmmol, transpiration rate. [file 1471-2229-14-81-S1.bmp]

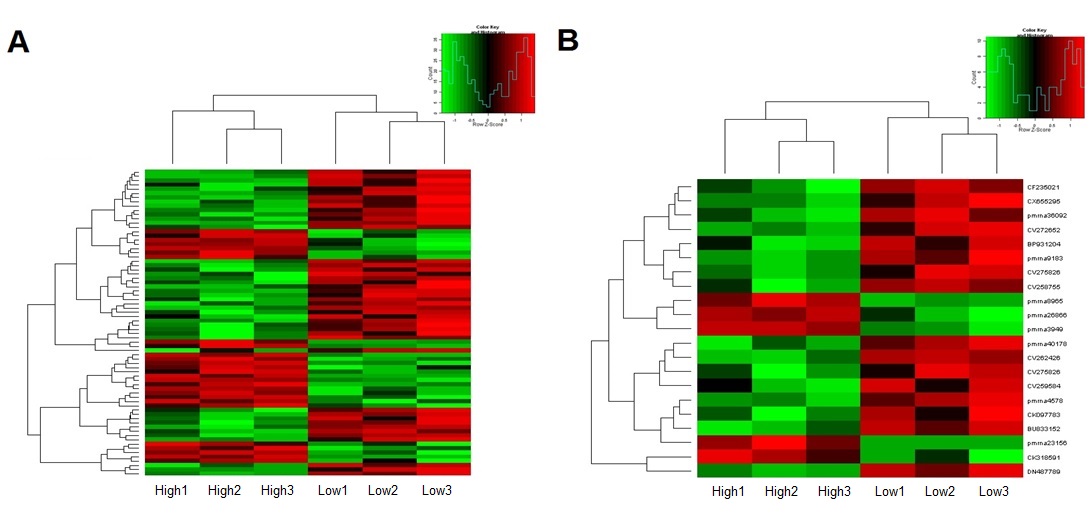

Supplement: Additional file 10: Figure S2 — Differentially expressed genes coordinating with organelles. Colors indicate up- (red) or down- (green) regulation. High1, High2, and High3 represent the pools of high photosynthetic rate plants and Low1, Low2, and Low3 represent the pools of low photosynthetic rate plants. In total, 72 differentially expressed genes related to organelles (chloroplast and/or mitochondrion) were identified. Of these, 27 genes were up-regulated and 45 were down-regulated in high photosynthetic rate gene pools. (A) Hierarchical clustering of differentially expressed genes coordinating with organelles. (B) Hierarchical clustering of differentially expressed genes involved with mitochondrial processes. [file 1471-2229-14-81-S10.jpeg]

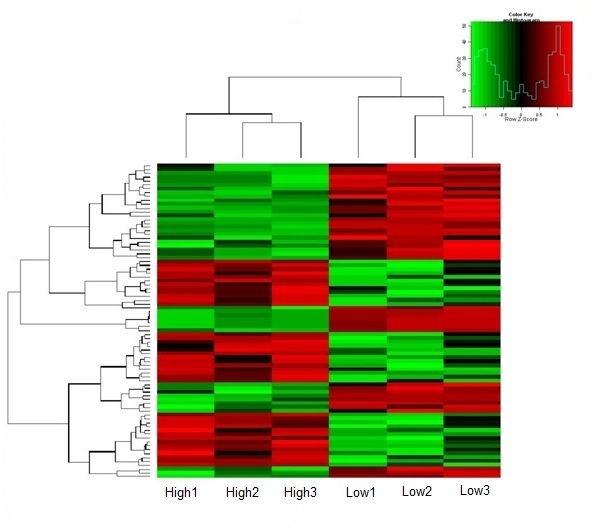

Supplement: Additional file 12: Figure S3 — Differentially expressed genes related to the cell wall. In total, 82 differentially expressed genes related to the plant cell wall were identified. Of these, 39 genes were up-regulated and 43 were down-regulated. (A) Hierarchical clustering of cell wall related genes. The number of up- and down-regulated genes is approximate. (B) Functional classification of cell wall related genes. There were clear differences between up- and down-regulated genes. 46.15% of the genes were grouped into metabolism and 38.46% genes related to response for stimulus. [file 1471-2229-14-81-S12.jpeg]
