# Supplementary material for: Identification and characterization of nuclear genes involved in photosynthesis in Populus
Source: BMC Plant Biol. 2014 Mar 27;14:81. doi: 10.1186/1471-2229-14-81 (PMC3986721; doi:10.1186/1471-2229-14-81)
Supplement: Additional file 2: Table S1 — Phenotypic data obtained from hybrids. [file 1471-2229-14-81-S2.doc]

**Table S1** Phenotypic data obtained from hybrids

| **Parameters Phenotype** | **Min** | **Max** | **Mean±SE** | **Standard Deviation** | **CV (%)** |
| --- | --- | --- | --- | --- | --- |
|
| Pn^a^ (μmol•m^–2^•s^–1^) | 2.295 | 25.159 | 13.360±0.116 | 3.863 | 28.915 |
| Cond^a^ (mol•m^–2^ •s^–1^) | 0.021 | 0.690 | 0.225±0.003 | 0.111 | 49.333 |
| Ci^a^ (μmol•mol^–1^) | 24.100 | 401.065 | 243.203±2.026 | 67.501 | 27.755 |
| Trmmol^a^(g•m^–2^•h^–1^) | 0.568 | 9.919 | 4.571±0.047 | 1.590 | 34.785 |
| Pn^b^ (μmol•m^–2^•s^–1^) | 3.465 | 21.980 | 13.551±0.200 | 3.459 | 25.526 |
| Cond^b^ (mol•m^–2^ •s^–1^) | 0.021 | 0.450 | 0.225±0.006 | 0.102 | 45.333 |
| Ci^b^ (μmol•mol^–1^) | 43.000 | 326.698 | 245.202±3.386 | 58.649 | 23.919 |
| Trmmol^b^ (g •m^–2^ •h^–1^) | 0.754 | 7.379 | 4.440±0.072 | 1.240 | 27.928 |
| Lignin content^b^ (%) | 7.087 | 16.219 | 10.510±0.085 | 1.475 | 14.082 |
| Holocellulose content^b^ (%) | 73.188 | 82.391 | 78.806±0.088 | 1.526 | 1.941 |
| α-cellulose content^b^ (%) | 23.860 | 46.094 | 37.168±0.196 | 3.403 | 9.147 |
| Microfiber angle^b^ (degree) | 11.265 | 21.506 | 14.826±0.086 | 1.486 | 10.047 |
| Tree height^b^ (m) | 0.750 | 3.990 | 2.476±0.037 | 0.633 | 25.403 |
| Diameter at breast height (1.3m)^b^ (cm) | 0.470 | 4.831 | 2.243±0.038 | 0.661 | 29.464 |
| Stem volume^b^ (m^3^) | 0.021×10^-3^ | 5.679×10^-3^ | 1.172×10^-3^±0.053×10^-3^ | 0.923×10^-3^ | 78.630 |

^a^There were three repetitions for each of the 1200 individual genotypes

^b^The phenotypic data is from association population (n=300; random selected from 1200 individual).

*Pn*, photosynthetic rate; *Cond*, conductance to H_2_O; *Ci*, intercellular CO_2_ concentration; *Trmmol*, transpiration rate; SE, standard error; CV, coefficient of phenotypic variation.
