# Supplementary material for: Identification and characterization of nuclear genes involved in photosynthesis in Populus
Source: BMC Plant Biol. 2014 Mar 27;14:81. doi: 10.1186/1471-2229-14-81 (PMC3986721; doi:10.1186/1471-2229-14-81)
Supplement: Additional file 3: Table S2 — Mean squares of ANOVA and broad heritabilities for the traits. [file 1471-2229-14-81-S3.doc]

| **Table S2 Mean squares of ANOVA and broad heritabilities for the traits** | | | | | |
| --- | --- | --- | --- | --- | --- |
| Source | Mean square | | | σ^2^g | h^2^(%) |
|  | Block | Genotypes | Error |  |  |
| df | 2 | 299 | 598 | - | - |
| Pn | 0.0009 | 35.8892 | 0.0032 | 11.9620 | 99.97 |
| Cond | 0.0001 | 0.0315 | 0.0001 | 0.0105 | 99.17 |
| Ci | 1.2555E+02 | 1.0319E+04 | 1.0015E+02 | 3.4063E+03 | 97.14 |
| Trm | 0.1241 | 4.6136 | 0.0292 | 1.5281 | 98.12 |
| Lig | 0.0004 | 6.5292 | 0.1719 | 2.1191 | 92.50 |
| Hol | 1.0535 | 6.9898 | 1.0337 | 1.9854 | 65.76 |
| α-cel | 0.6195 | 34.7467 | 2.4238 | 10.7743 | 81.64 |
| MFA | 0.1797 | 6.6247 | 0.3673 | 2.0858 | 85.03 |
| H | 0.0064 | 1.2035 | 0.0104 | 0.3977 | 97.45 |
| D | 0.0005 | 1.3111 | 0.0078 | 0.4344 | 98.24 |
| V | 2.56E-06 | 4.82E-09 | 1.26E-08 | 8.49E-07 | 98.53 |
| Pn, photosynthetic rate; Cond, conductance to H_2_O; Ci, intercellular CO_2_ concentration; Trm, transpiration rate; Lig, lignin content; Hol, holocellulose; α-cel, α-cellulose; MFA, microfiber angle; H, tree height; D, diameter at chest height; V, stem volume; σ^2^g, genetic variance; h^2^, broad sense heritability. | | | | | |
