# Supplementary material for: Identification and characterization of nuclear genes involved in photosynthesis in Populus
Source: BMC Plant Biol. 2014 Mar 27;14:81. doi: 10.1186/1471-2229-14-81 (PMC3986721; doi:10.1186/1471-2229-14-81)
Supplement: Additional file 6: Table S3 — Primers used for real-time PCR analysis. [file 1471-2229-14-81-S6.doc]

| **Table S3 Primers used for real-time PCR analysis** | | | | |
| --- | --- | --- | --- | --- |
|  | **Gene** | **Suggested function** | **Forward primer**  **(5'→3')** | **Reverse primer**  **(5'→3')** |
| Up-  regulated | CF936190RT | Polyketide cyclase/dehydrase and lipid transport superfamily protein | CCCTTAGCACGGAGGAATAGAA | CTGGCATGATCACAGACATGTG |
|  | AF515607RT | Xyloglucan endotransglycosylase precursor (XET16A) | GGTCCAAGGCACCGTTCAT | AGGCCTCGCACCCATCTAT |
|  | CK089075RT | Stress responsive A/B Barrel domain-containing protein | AGGCCCGGAGATGCTTACA | CCTTCTTGTCAAACGTCATGGA |
|  | CV273041RT | Gibberellin-regulated protein (GASA) | TGGGACTTATGGGAACAAGCA | GTTTGCCCTTGGAGTTCTTCAT |
|  | CV260219RT | Extensin | ACCACTTTTTGTGGATTGCAGAA | TTTCAGGTTGGCCTCTACCAA |
| Reference gene | | Actin | CTCCATCATGAAATGCGATG | TTGGGGCTAGTGCTGAGATT |
| Down-  regualted | AJ780277RT | PR-6 proteinase inhibitor family | CATGGCCAGAGCTTCTTGGA | TCCACAAGAGGATTTTCTCTCTCA |
|  | CX183751RT | Heat shock transcription factor family protein | TAATTCAAGTCGCACCACAAGAG | CGGACGATGATAAAGTGGATGA |
|  | CX185631RT | Putative beta-1,4-endoglucanase | GAAAGGAGGTACGCCGAAGA | TTCATCGAACATGGCAAATATGTA |
|  | CV260015RT | Aldolase-type TIM barrel family protein | GCAGGCTGCCCTAGTGTGA | TCTGTCGCGGTCAGTCCTTAC |
|  | DN487027RT | Heme oxygenase | CCATGGCCTTGTTATCCTTGA | GGACAAGAGGCTCCATTGATG |
| Reference gene | | Actin | CTCCATCATGAAATGCGATG | TTGGGGCTAGTGCTGAGATT |
