# Supplementary material for: Identification and characterization of nuclear genes involved in photosynthesis in Populus
Source: BMC Plant Biol. 2014 Mar 27;14:81. doi: 10.1186/1471-2229-14-81 (PMC3986721; doi:10.1186/1471-2229-14-81)
Supplement: Additional file 7: Table S4 — The result of t-test analysis. [file 1471-2229-14-81-S7.doc]

Table S4 The result of t-test analysis

|  | Method | Gene Pool | Number | Mean | Std.Error Mean | *P*-value. | Mean difference | Std.Error difference |
| --- | --- | --- | --- | --- | --- | --- | --- | --- |
| CF936190 | Microarray^a^ | High *Pn* group | 3 | 9.462 | 1.080 | 0.049 | 4.328 | 1.119 |
|  |  | Low *Pn* group | 3 | 5.133 | 0.295 |  |  |  |
|  | Real time-PCR^b^ | High *Pn* group | 3 | 1.074 | 0.423 | 0.078 | 1.006 | 0.427 |
|  |  | Low *Pn* group | 3 | 0.067 | 0.052 |  |  |  |
|  | Individuals^c^ | High *Pn* group | 15 | 0.870 | 0.177 | 0.010 | 0.559 | 0.197 |
|  |  | Low *Pn* group | 15 | 0.311 | 0.086 |  |  |  |
| AF515607 | Microarray^a^ | High *Pn* group | 3 | 11.105 | 0.476 | 0.009 | 3.512 | 0.732 |
|  |  | Low *Pn* group | 3 | 7.592 | 0.556 |  |  |  |
|  | Real time-PCR^b^ | High *Pn* group | 3 | 1.254 | 0.156 | 0.009 | 0.905 | 0.192 |
|  |  | Low *Pn* group | 3 | 0.349 | 0.113 |  |  |  |
|  | Individuals^c^ | High *Pn* group | 15 | 0.856 | 0.196 | 0.003 | 0.705 | 0.200 |
|  |  | Low *Pn* group | 15 | 0.151 | 0.037 |  |  |  |
| CK089075 | Microarray^a^ | High *Pn* group | 3 | 10.102 | 0.629 | 0.013 | 3.008 | 0.711 |
|  |  | Low *Pn* group | 3 | 7.093 | 0.333 |  |  |  |
|  | Real time-PCR^b^ | High *Pn* group | 3 | 0.900 | 0.195 | 0.040 | 0.607 | 0.202 |
|  |  | Low *Pn* group | 3 | 0.292 | 0.052 |  |  |  |
| CV273041 | Microarray^a^ | High *Pn* group | 3 | 9.909 | 0.526 | 0.046 | 2.014 | 0.703 |
|  |  | Low *Pn* group | 3 | 7.896 | 0.466 |  |  |  |
|  | Real time-PCR^b^ | High *Pn* group | 3 | 0.737 | 0.202 | 0.037 | 0.629 | 0.204 |
|  |  | Low *Pn* group | 3 | 0.107 | 0.031 |  |  |  |
| CV260219 | Microarray^a^ | High *Pn* group | 3 | 9.409 | 0.285 | 0.041 | 1.519 | 0.510 |
|  |  | Low *Pn* group | 3 | 7.890 | 0.423 |  |  |  |
|  | Real time-PCR^b^ | High *Pn* group | 3 | 0.414 | 0.103 | 0.104 | 0.293 | 0.104 |
|  |  | Low *Pn* group | 3 | 0.121 | 0.009 |  |  |  |
| AJ780277 | Microarray^a^ | High *Pn* group | 3 | 3.825 | 0.787 | 0.007 | -5.124 | 0.999 |
|  |  | Low *Pn* group | 3 | 8.950 | 0.616 |  |  |  |
|  | Real time-PCR^b^ | High *Pn* group | 3 | 0.026 | 0.007 | 0.002 | -0.205 | 0.030 |
|  |  | Low *Pn* group | 3 | 0.232 | 0.029 |  |  |  |
| CX183751 | Microarray^a^ | High *Pn* group | 3 | 3.445 | 0.977 | 0.013 | -4.218 | 0.991 |
|  |  | Low *Pn* group | 3 | 7.663 | 0.171 |  |  |  |
|  | Real time-PCR^b^ | High *Pn* group | 3 | 0.052 | 0.006 | 0.043 | -0.351 | 0.120 |
|  |  | Low *Pn* group | 3 | 0.403 | 0.119 |  |  |  |
| CX185631 | Microarray^a^ | High *Pn* group | 3 | 9.047 | 0.688 | 0.016 | -3.796 | 0.952 |
|  |  | Low *Pn* group | 3 | 12.843 | 0.657 |  |  |  |
|  | Real time-PCR^b^ | High *Pn* group | 3 | 0.345 | 0.023 | 0.044 | -2.541 | 0.552 |
|  |  | Low *Pn* group | 3 | 2.887 | 0.551 |  |  |  |
|  | Individuals^c^ | High *Pn* group | 15 | 0.430 | 0.124 | <0.001 | -3.138 | 0.680 |
|  |  | Low *Pn* group | 15 | 3.568 | 0.668 |  |  |  |
| CV260015 | Microarray^a^ | High *Pn* group | 3 | 7.877 | 0.156 | 0.001 | -1.620 | 0.202 |
|  |  | Low *Pn* group | 3 | 9.498 | 0.128 |  |  |  |
|  | Real time-PCR^b^ | High *Pn* group | 3 | 0.123 | 0.011 | 0.050 | -0.706 | 0.254 |
|  |  | Low *Pn* group | 3 | 0.829 | 0.253 |  |  |  |
|  | Individuals^c^ | High *Pn* group | 15 | 0.171 | 0.038 | 0.005 | -0.499 | 0.154 |
|  |  | Low *Pn* group | 15 | 0.670 | 0.149 |  |  |  |
| DN487027 | Microarray^a^ | High *Pn* group | 3 | 7.970 | 0.168 | 0.012 | -1.189 | 0.273 |
|  |  | Low *Pn* group | 3 | 9.159 | 0.215 |  |  |  |
|  | Real time-PCR^b^ | High *Pn* group | 3 | 0.121 | 0.013 | 0.048 | -0.242 | 0.086 |
|  |  | Low *Pn* group | 3 | 0.363 | 0.085 |  |  |  |
| a, The analysis between high *Pn* (High1, High2 and High3) and Low *Pn* groups (Low1, Low2 and Low3) utilizing t-test with data from microarray analysis. | | | | | | | | |
| b, The analysis between high *Pn* (High1, High2 and High3) and Low *Pn* groups (Low1, Low2 and Low3) utilizing t-test with data from real time-PCR, for which the template was the mixed cDNA of BSA pools that the samples for microarray analysis. | | | | | | | | |
| c, The analysis between high *Pn* (High1, High2 and High3) and Low *Pn* groups (Low1, Low2 and Low3) utilizing t-test with data from real time-PCR, for which the template were from the individuals used for constructing BSA pools. | | | | | | | | |
| Std.Error, standard error | | | | | | | | |
