# Supplementary material for: Identification and characterization of nuclear genes involved in photosynthesis in Populus
Source: BMC Plant Biol. 2014 Mar 27;14:81. doi: 10.1186/1471-2229-14-81 (PMC3986721; doi:10.1186/1471-2229-14-81)
Supplement: Additional file 8: Table S5 — Gene ontology enrichment analysis for the differentially expressed genes. [file 1471-2229-14-81-S8.doc]

**Table S5 Gene ontology enrichment analysis for the differentially expressed genes**

| **Pattern** | **GO term** | **Ontology** | **Description** | **p-value** | **FDR** |
| --- | --- | --- | --- | --- | --- |
| Down-  regulated | GO:0008152 | P | metabolic process | 5.50E-06 | 0.0014 |
|  | GO:0003824 | F | catalytic activity | 4.40E-07 | 0.00013 |
|  | GO:0043565 | F | sequence-specific DNA binding | 9.20E-06 | 0.0013 |
|  | GO:0004857 | F | enzyme inhibitor activity | 1.50E-05 | 0.0014 |
|  | GO:0016491 | F | oxidoreductase activity | 2.20E-05 | 0.0016 |
|  | GO:0004866 | F | endopeptidase inhibitor activity | 4.20E-05 | 0.002 |
|  | GO:0030414 | F | peptidase inhibitor activity | 4.20E-05 | 0.002 |
|  | GO:0008238 | F | exopeptidase activity | 5.20E-05 | 0.0021 |
|  | GO:0016787 | F | hydrolase activity | 9.30E-05 | 0.003 |
|  | GO:0003700 | F | transcription factor activity | 8.90E-05 | 0.003 |
|  | GO:0004553 | F | hydrolase activity, hydrolyzing O-glycosyl compounds | 4.90E-04 | 0.012 |
|  | GO:0030246 | F | carbohydrate binding | 4.80E-04 | 0.012 |
|  | GO:0030528 | F | transcription regulator activity | 0.00042 | 0.012 |
|  | GO:0030234 | F | enzyme regulator activity | 0.00062 | 0.014 |
|  | GO:0016798 | F | hydrolase activity, acting on glycosyl bonds | 0.00076 | 0.016 |
|  | GO:0004713 | F | protein tyrosine kinase activity | 0.0015 | 0.025 |
|  | GO:0016684 | F | oxidoreductase activity, acting on peroxide as acceptor | 0.0015 | 0.025 |
|  | GO:0004601 | F | peroxidase activity | 0.0015 | 0.025 |
|  | GO:0005506 | F | iron ion binding | 2.20E-03 | 3.50E-02 |
|  | GO:0020037 | F | heme binding | 2.70E-03 | 3.70E-02 |
|  | GO:0046906 | F | tetrapyrrole binding | 2.70E-03 | 3.70E-02 |
|  | GO:0005215 | F | transporter activity | 2.60E-03 | 3.70E-02 |
|  | GO:0016209 | F | antioxidant activity | 3.40E-03 | 4.40E-02 |
| Up-  regulated | GO:0030312 | C | external encapsulating structure | 6.00E-07 | 0.000015 |
|  | GO:0005618 | C | cell wall | 2.90E-06 | 0.000037 |
| P, Biological process; F, Molecular function; C, Cell component; FDR, false discovery rate. | | | | | |
