# Supplementary material for: Identification and characterization of nuclear genes involved in photosynthesis in Populus
Source: BMC Plant Biol. 2014 Mar 27;14:81. doi: 10.1186/1471-2229-14-81 (PMC3986721; doi:10.1186/1471-2229-14-81)
Supplement: Additional file 16: Table S6 — The annotation of candidate genes for SNP analysis. [file 1471-2229-14-81-S16.doc]

**Table S6 The annotation of candidate genes for SNP analysis**

| **Gene** | **ID** | **Phytozome ATG** | **Public ID** | **Suggested Function** | **FC value** |
| --- | --- | --- | --- | --- | --- |
| XET | POPTR_0003s15800 | AT5G13870 | AF515607 | Xyloglucan endotransglycosylase precursor | 11.41 |
| Dabb | POPTR_0009s15030 | AT5G22580 | CK089075 | stress responsive A/B Barrel domain-containing protein | 8.05 |
| GASA | POPTR_0014s02030.1 | AT5G59845 | CV273041 | gibberellin-regulated protein | 4.04 |
| SAUR | POPTR_0009s12890.1 | AT1G75580 | CV251026 | SAUR family protein | 2.94 |
| CGSS | POPTR_0003s18610.1 | AT1G64660 | CK115458 | cystathionine gamma-synthase | 47.52 |
| PI | POPTR_0010s08600 | AT2G38870 | AJ780277 | PR-6 proteinase inhibitor family | 34.88 |

FC, fold change.
