# Supplementary material for: Identification and characterization of nuclear genes involved in photosynthesis in Populus
Source: BMC Plant Biol. 2014 Mar 27;14:81. doi: 10.1186/1471-2229-14-81 (PMC3986721; doi:10.1186/1471-2229-14-81)
Supplement: Additional file 17: Table S7 — Number and distribution of SNPs detected in this study. [file 1471-2229-14-81-S17.doc]

**Table S7 Number and distribution of SNPs detected in this study**

| **Gene** | **Quantity** | **Frequency** | **Common SNP** | **5'UTR** | **Exon** | **Intron** | **3'UTR** |
| --- | --- | --- | --- | --- | --- | --- | --- |
| XET | 160 | 14 | 54 | 6 | 18 | 27 | 3 |
| Dabb | 19 | 34 | 9 | 0 | 6 | 0 | 3 |
| GASA | 42 | 15 | 21 | 1 | 6 | 7 | 7 |
| SAUR | 19 | 36 | 5 | 0 | 4 | 0 | 1 |
| CGSS | 68 | 27 | 24 | 0 | 23 | 1 | 0 |
| PI | 67 | 7 | 45 | 13 | 16 | 13 | 3 |
| Common SNP, minor allele frequency ≥10% | | | | | | | |
