# Supplementary material for: Identification and characterization of nuclear genes involved in photosynthesis in Populus
Source: BMC Plant Biol. 2014 Mar 27;14:81. doi: 10.1186/1471-2229-14-81 (PMC3986721; doi:10.1186/1471-2229-14-81)
Supplement: Additional file 18 — Experimental data of 1738 (158 SNPs × 11 traits) single-marker analyses to account for linear regression by single factor ANOVA (n = 300). [file 1471-2229-14-81-S18.doc]

**Table S8 Comparison with 1590 genes contained in the 23 regulons**

| **Gene model^a^** | **AGI accession^b^** | **Regulon^c^** | **Subcellular Location^c^** | **Description** |
| --- | --- | --- | --- | --- |
| POPTR_0015s14670 | AT5G62360.1 | 13 | other | invertasepectin methylesterase inhibitor family protein |
| POPTR_0004s19420 | AT2G17120.1 | 7 | other | peptidoglycan-binding LysM domain-containing protein |
| POPTR_0018s05360 | AT3G14310.1 | 11 | other | Pectinesterase |
| POPTR_0004s19420 | AT2G17120.1 | 7 | other | peptidoglycan-binding LysM |
| POPTR_0022s00220 | AT3G14310.1 | 11 | other | putative pectin methylesterase |
| POPTR_0003s07040 | AT3G14310.1 | 11 | other | pectinesterase |
| POPTR_0010s20900 | AT2G39710.1 | 21 | other | Aspartic proteinase nepenthesin-1 |
| POPTR_0009s08540 | AT1G09970.1 | 17 | other | leucine-rich receptor-like protein kinase |
| POPTR_0007s09490 | AT4G15560.1 | 2 | Chl | 1-deoxy-D-xylulose 5-phosphate synthase |
| POPTR_0015s12150 | AT5G51070.1 | 21 | Chl | chloroplast precursor |
| POPTR_0001s47450 | AT1G60950.1 | 1 | Chl | Ferredoxin |
| POPTR_0004s20660 | AT1G74470.1 | 9 | Chl | geranylgeranyl reductase |
| POPTR_0002s02190 | AT4G38660.1 | 11 | other | pathogenesis-related thaumatin family protein |
| POPTR_0011s06990 | AT1G29280.1 | 12 | other | WRKY transcription factor |
| POPTR_0018s00970 | AT4G32650.1 | 17 | Mit | Potassium channel KAT3 |
| POPTR_0001s19030 | AT1G59870.1 | 18 | Chl | ABC transporter family protein |
| POPTR_0018s02140 | AT5G11090.1 | 12 | Mit | serine-rich protein-like protein |
| POPTR_0009s16900 | AT2G17230.1 | 15 | other | phosphate-responsive 1 family protein |
| POPTR_0008s00540 | AT1G51080.1 | 5 | Chl | unknown |
| POPTR_0009s16300 | AT5G06270.1 | 12 | Mit | unknown |

^a^, Gene model was derived from the Joint Genome Institute Web site (JGI)(<http://www.jgi.doe.gov/>); [^b^, ATG accession was derived from TAIR (http://www.arabidopsis.org)](http://www.arabidopsis.org/); ^c^, Data from Biehl et. al (2005)
