# Supplementary material for: Identification and characterization of nuclear genes involved in photosynthesis in Populus
Source: BMC Plant Biol. 2014 Mar 27;14:81. doi: 10.1186/1471-2229-14-81 (PMC3986721; doi:10.1186/1471-2229-14-81)
Supplement: Additional file 21: Table S9 — Growing conditions in Xiao Tangshan horticulture fields. [file 1471-2229-14-81-S21.doc]

| **Table S9 Growing condition in Xiao Tangshan horticulture fields** | | | | | |
| --- | --- | --- | --- | --- | --- |
| Data | Average of temperature (℃) | Maximal temperature (℃) | Minimum temperature (℃) | Relative humidity | Hours of sunshine (h) |
| 7.20 | 21.7 | 27.9 | 16.7 | 78% | 9.7 |
| 7.21 | 22.2 | 26.8 | 19.1 | 76% | 12.0 |
| 7.22 | 22.7 | 29.6 | 17.0 | 75% | 11.8 |
| 7.23 | 21.9 | 30.9 | 17.4 | 71% | 12.7 |
| 7.24 | 23.6 | 30.2 | 16.1 | 77% | 9.3 |
| 7.25 | 24.0 | 29.3 | 19.0 | 81% | 6.7 |
| 7.26 | 19.4 | 23.4 | 15.2 | 60% | 12.7 |
| 7.27 | 17.2 | 22.2 | 13.4 | 67% | 6.0 |
| 7.28 | 19.8 | 27.1 | 11.7 | 72% | 11.6 |
| 7.29 | 21.9 | 28.3 | 15.6 | 70% | 10.8 |
| 7.30 | 20.7 | 23.6 | 18.4 | 71% | 7.1 |
| 7.31 | 18.0 | 20.1 | 16.2 | 74% | 3.5 |
| 8.1 | 17.5 | 22.4 | 12.8 | 61% | 12.2 |
| 8.2 | 19.3 | 25.2 | 11.7 | 60% | 13.0 |
| 8.3 | 17.5 | 23.4 | 11.4 | 77% | 4.0 |
| 8.4 | 15.9 | 19.1 | 15.1 | 84% | 3.1 |
| 8.5 | 15.6 | 20.9 | 9.2 | 86% | 2.3 |
| 8.6 | 18.3 | 25.3 | 11.6 | 69% | 13.3 |
| 8.7 | 19.0 | 27.5 | 11.4 | 67% | 8.6 |
| 8.8 | 20.2 | 26.5 | 13.1 | 78% | 10.6 |
