# Supplementary material for: Identification and characterization of nuclear genes involved in photosynthesis in Populus
Source: BMC Plant Biol. 2014 Mar 27;14:81. doi: 10.1186/1471-2229-14-81 (PMC3986721; doi:10.1186/1471-2229-14-81)
Supplement: Additional file 22: Table S10 — T-test analysis of BSA pools (n = 30). [file 1471-2229-14-81-S22.doc]

**Table S10 T-test analysis of BSA pools (n=30)**

| Number | Mean±SE | Mean±SE | Mean difference | Std. Error.difference | *P*-value |
| --- | --- | --- | --- | --- | --- |
| High1 | 22.948±0.899 | 22.137±0.461 | 12.207 | 0.817 | <0.001 |
| High2 | 21.164±0.687 |
| High3 | 22.298±0.740 |
| Low1 | 9.258±1.278 | 9.930±0.675 |
| Low2 | 10.986±0.797 |
| Low3 | 9.544±1.440 |

In total thirty samples were segregated into two groups (high and low *Pn*) producing three biological repeats for each group named High1, High2, High3, Low1, Low2 and Low3. And there are 5 individuals in each repeat.
